# Impaired glycine neurotransmission causes adolescent idiopathic scoliosis

**DOI:** 10.1172/JCI168783

**Published:** 2024-01-16

**Authors:** Xiaolu Wang, Ming Yue, Jason Pui Yin Cheung, Prudence Wing Hang Cheung, Yanhui Fan, Meicheng Wu, Xiaojun Wang, Sen Zhao, Anas M. Khanshour, Jonathan J. Rios, Zheyi Chen, Xiwei Wang, Wenwei Tu, Danny Chan, Qiuju Yuan, Dajiang Qin, Guixing Qiu, Zhihong Wu, Terry Jianguo Zhang, Shiro Ikegawa, Nan Wu, Carol A. Wise, Yong Hu, Keith Dip Kei Luk, You-Qiang Song, Bo Gao

**Affiliations:** 1 Department of Orthopaedics and Traumatology, School of Clinical Medicine, Li Ka Shing Faculty of Medicine, University of Hong Kong, Pokfulam, Hong Kong, China.; 2 School of Biomedical Sciences, Faculty of Medicine, Chinese University of Hong Kong, Shatin, Hong Kong, China.; 3 School of Biomedical Sciences, Li Ka Shing Faculty of Medicine, University of Hong Kong, Pokfulam, Hong Kong, China.; 4 Department of Orthopaedics and Traumatology, University of Hong Kong–Shenzhen Hospital, Shenzhen, China.; 5 Department of Orthopaedic Surgery, Department of Medical Research Center, Key Laboratory of Big Data for Spinal Deformities, State Key Laboratory of Complex Severe and Rare Diseases, Beijing Key Laboratory for Genetic Research of Skeletal Deformity, Peking Union Medical College Hospital (PUMCH) and Chinese Academy of Medical Sciences, Beijing, China.; 6 Center for Pediatric Bone Biology and Translational Research, Scottish Rite for Children (SRC), Dallas, Texas, USA.; 7 Eugene McDermott Center for Human Growth and Development, Departments of Orthopaedic Surgery and Pediatrics, University of Texas Southwestern Medical Center, Dallas, Texas, USA.; 8 Department of Paediatrics and Adolescent Medicine, School of Clinical Medicine, Li Ka Shing Faculty of Medicine, University of Hong Kong, Pokfulam, Hong Kong, China.; 9 Centre for Regenerative Medicine and Health, Hong Kong Institute of Science & Innovation, Chinese Academy of Sciences, Tai Po, Hong Kong, China.; 10 Laboratory of Bone and Joint Diseases, RIKEN Center for Integrative Medical Sciences, Tokyo, Japan.; 11 Department of Medicine, University of Hong Kong–Shenzhen Hospital, Shenzhen, China.; 12 State Key Laboratory of Brain and Cognitive Sciences, University of Hong Kong, Pokfulam, Hong Kong, China.; 13 Centre for Translational Stem Cell Biology, Tai Po, Hong Kong, China.; 14 Key Laboratory of Regenerative Medicine, Ministry of Education, School of Biomedical Sciences, Faculty of Medicine, Chinese University of Hong Kong, Shatin, Hong Kong, China.

**Keywords:** Bone Biology, Genetics, Bone disease, Genetic diseases, Orthopedics

## Abstract

Adolescent idiopathic scoliosis (AIS) is the most common form of spinal deformity, affecting millions of adolescents worldwide, but it lacks a defined theory of etiopathogenesis. Because of this, treatment of AIS is limited to bracing and/or invasive surgery after onset. Preonset diagnosis or preventive treatment remains unavailable. Here, we performed a genetic analysis of a large multicenter AIS cohort and identified disease-causing and predisposing variants of *SLC6A9* in multigeneration families, trios, and sporadic patients. Variants of *SLC6A9*, which encodes glycine transporter 1 (GLYT1), reduced glycine-uptake activity in cells, leading to increased extracellular glycine levels and aberrant glycinergic neurotransmission. *Slc6a9* mutant zebrafish exhibited discoordination of spinal neural activities and pronounced lateral spinal curvature, a phenotype resembling human patients. The penetrance and severity of curvature were sensitive to the dosage of functional glyt1. Administration of a glycine receptor antagonist or a clinically used glycine neutralizer (sodium benzoate) partially rescued the phenotype. Our results indicate a neuropathic origin for “idiopathic” scoliosis, involving the dysfunction of synaptic neurotransmission and central pattern generators (CPGs), potentially a common cause of AIS. Our work further suggests avenues for early diagnosis and intervention of AIS in preadolescents.

## Abstract 2

Genetic variants affecting synaptic neurotransmission and central pattern generators are strong causal risk factors of adolescent idiopathic scoliosis.

## Graphical abstract

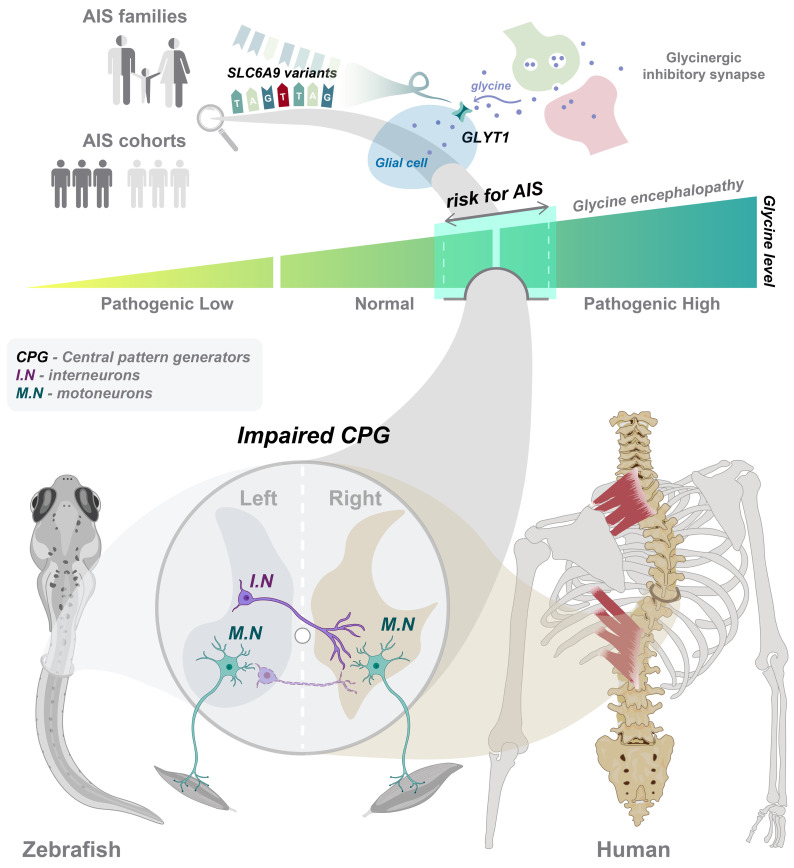

## Introduction

Adolescent idiopathic scoliosis (AIS) is a condition in which the spine is deformed with a lateral curvature exceeding 10 degrees in otherwise healthy adolescents (1, 2). With a prevalence of 0.47% to 5.2% in adolescents worldwide, AIS is the most common pediatric skeletal disorder and usually worsens during the pubertal growth spurt (1–5). In severe cases, AIS can cause cardiopulmonary difficulties, leading to shortness of breath and potential mortality (2, 6–9). Despite its high prevalence and long-term physical and mental health implications, AIS lacks an agreed-upon theory of etiopathogenesis, which severely impedes the rational development of early diagnostic, preventive, and therapeutic strategies (1, 2, 4). Although the causes of AIS are believed to be multifactorial, population and twin studies suggest a strong contribution of genetic factors to the development of AIS (10–12). Many common or rare variants in coding or noncoding regions of genes (e.g., *LBX1*, *GPR126*, *PAX1*, *CHL1*, *POC5*) have been identified as being associated with AIS (13–19). However, the vast majority of the heritability of AIS is still unexplained, and causative mechanisms linking the susceptible genes to AIS remain unclear.

Glycine is a crucial neurotransmitter that plays a role in both inhibitory and excitatory neurotransmission in the CNS (20). In the spinal cord and brain stem, glycine mainly acts as an inhibitor by binding to ionotropic glycine receptors (GlyRs), leading to postsynaptic hyperpolarization and inhibition of neural activities. Extracellular glycine levels are tightly controlled by 2 glycine transporters, glycine transporter 1 (GLYT1) and GLYT2. GLYT1 is primarily expressed by astrocytes adjacent to glycinergic neurons to facilitate the rapid clearance of glycine from the synaptic cleft (Supplemental Figure 1; supplemental material available online with this article; https://doi.org/10.1172/JCI168783DS1) (21–23). Homozygous mutations in the *SLC6A9* gene that encodes GLYT1 cause glycine encephalopathy, also known as nonketotic hyperglycinemia (NKH), which is a severe neurological disease caused by abnormally high levels of glycine in the cerebrospinal fluid (CSF) and characterized by respiratory failure, progressive hypotonia, and startle-like reflexes (24, 25). Interestingly, although most glycine encephalopathy patients die before 7 months of age, those who survive show progressive early onset scoliosis as a result of apparent neurological defects (26–28).

In this study, we investigate the genetic basis and pathogenic mechanism of AIS in a multicenter cohort of patients. Linkage analysis and genome sequencing identified a number of rare heterozygous variants in *SLC6A9*, which were mostly deleterious, affecting the membrane presentation and glycine uptake of GLYT1. The AIS patients exhibited increased plasma glycine levels and aberrant paraspinal muscle activities. In the zebrafish model, disruption of *slc6a9* led to an AIS-like phenotype. We showed that disturbance in the normal function of central pattern generators (CPGs) by either excessive glycine or developmental defects resulted in lateral spinal curvature. We further tested the feasibility of treating this deficiency to prevent scoliosis.

## Results

### Identification of SLC6A9 variants in AIS patients.

**Figure 1:**
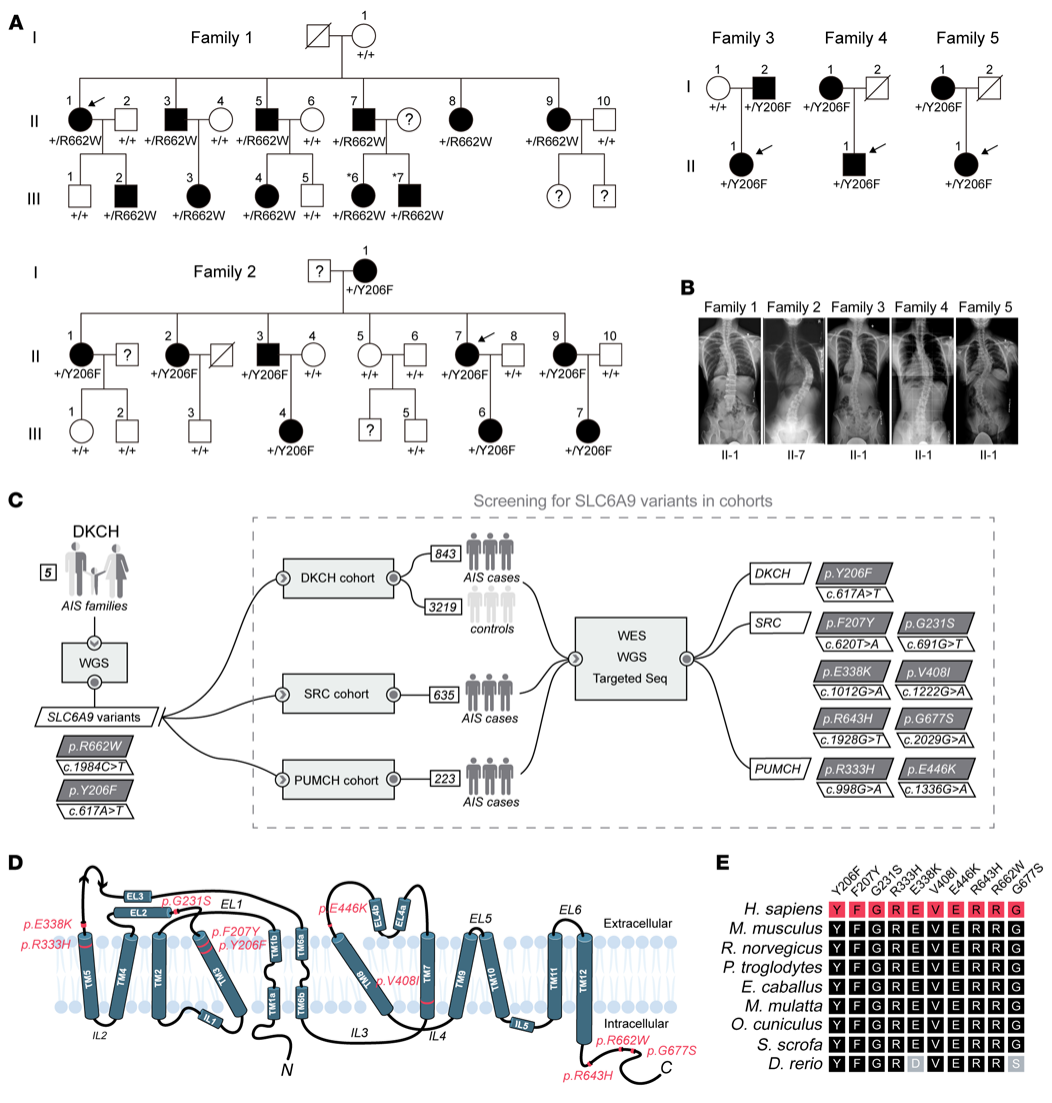
Heterozygous missense variants in *SLC6A9* leading to AIS. (**A**) Pedigree of 5 AIS families with dominant inheritance. Squares and circles denote male and female family members, respectively. Filled and open symbols represent affected and unaffected family members, respectively. Individuals marked with numbers indicate the family members recruited in this study. Question marks and diagonal slashes indicate unavailable and deceased members, respectively. The term +/+ denotes WT, and +/R662W or +/Y206F denotes heterozygous variant of *SLC6A9*. Arrows indicate the probands. (**B**) Spinal radiographs of the probands of family 1-5. (**C**) Workflow for the identification of *SLC6A9* variants in the multicenter AIS cohort. The number of families and sporadic patients enrolled from each involved hospital is indicated, and the identified *SLC6A9* variants are highlighted in the oblique boxes. (**D**) Membrane topological features of GLYT1. Positions of the identified variants are indicated in the diagram. TM, transmembrane; IL, intracellular loop; EL, extracellular loop. N and C indicate the N- and C-termini of GLYT1, respectively. (**E**) Evolutionary conservation of altered GLYT1 amino acids associated with AIS. Each variant is shown on the top.

We performed a genetic analysis to identify pathogenic variants in a multicenter AIS cohort consisting of multigeneration families, trios, and approximately 1,700 sporadic patients. We first conducted whole-genome sequencing (WGS) on 10 individuals in family 1 (II-1, II-2, II-3, II-5, II-7, II-8, III-1, III-2, III-3, and III-4) and 12 individuals in family 2 (I-1, II-1, II-2, II-3, II-4, II-5, II-7, II-8, II-9, III-3, III-4, and III-6) (Figure 1, A and B, Supplemental Figure 2, and Supplemental Table 1). Filtering the detected variants identified 3 candidate genes in each of the 2 families, which were all located within a short interval of chromosome 1p (Supplemental Figure 3A and Supplemental Table 2). Linkage analysis of the 2 families also revealed a unique linkage region located on chromosome 1p34.1, with a maximum logarithm of odds (LOD) score greater than 3.0 (Supplemental Figure 3B). Intriguingly, each family had a nonsynonymous coding variant (c.1984C>T, p.R662W in family 1 and c.617A>T, p.Y206F in family 2) in *SLC6A9* (NM_201649.4; NP_964012.2), which located in the linkage locus and segregated with the phenotype (Figure 1A and Supplemental Table 2). These 2 variants were very rare in the Genome Aggregation Database (https://gnomad.broadinstitute.org/) (gnomAD v3.1.2) (p.Y206F, 1.449 × 10^–4^; p.R662W, 6.571 × 10^–6^), and the amino acid substitutions were predicted to be deleterious or damaging by multiple algorithms (Supplemental Table 2). The results from these 2 families indicate that *SLC6A9* is a potential causal gene for AIS.

We screened potential *SLC6A9* variants in 3 sporadic AIS genome sequencing data sets consisting of 118 patients from the Duchess of Kent Children’s Hospital (DKCH, Hong Kong, China), 223 patients from PUMCH, and 635 patients from SRC. In the DKCH cohort, we identified 5 individuals harboring the same heterozygous *SLC6A9* variant (c.617A>T, p.Y206F), which was first identified in family 2 (Supplemental Table 1). Further clinical and genetic investigations revealed that the parents of 3 of the patients were also affected and carried the c.617A>T, p.Y206F variant (family 3-5) (Figure 1A and Supplemental Table 1). We also identified several rare heterozygous missense variants of *SLC6A9* in the PUMCH (2 variants in 2 patients) and SRC (6 variants in 12 patients) cohorts (Figure 1C and Supplemental Table 1). All these variants altered highly conserved residues in various regions of GLYT1, the majority of which were predicted to be deleterious or damaging by multiple algorithms (Figure 1, D and E, and Supplemental Table 3).

We next performed targeted sequencing of *SLC6A9* in the cohort of 725 sporadic AIS patients and a cohort of 3,219 ethnicity-matched participants without AIS in Hong Kong. We identified 9 out of 725 AIS patients and 7 out of 3,219 controls as harboring the c.617A>T, p.Y206F variant, whereas the c.1984C>T, p.R662W variant that was detected in family 1 was not found in any of sporadic patients or controls. The p.Y206F variant had a total allele frequency of 0.884% (15/1,696) in the Hong Kong AIS cohort (11 sporadic and 4 familial alleles out of 840 sporadic patients and 8 families), which was significantly higher compared with the local non-AIS controls (0.109%, 7/6,438, *P* = 2.39 × 10^–6^), Chinese general population (29, 30) (0.280%, *P* = 2.74 × 10^–4^), and data in gnomAD (0.014%, *P* = 3.56 × 10^–20^) (Supplemental Tables 4 and 5). Notably, it is unclear whether the individuals included in the latter 2 data sets were examined for scoliosis. The identification of multiple rare variants in familial and sporadic patients and the strong association of the p.Y206F variant with AIS further indicate the genetic susceptibility of *SLC6A9* to AIS.

### Plasma glycine levels and aberrant paraspinal muscle activity in SLC6A9 variant carriers.

**Figure 2:**
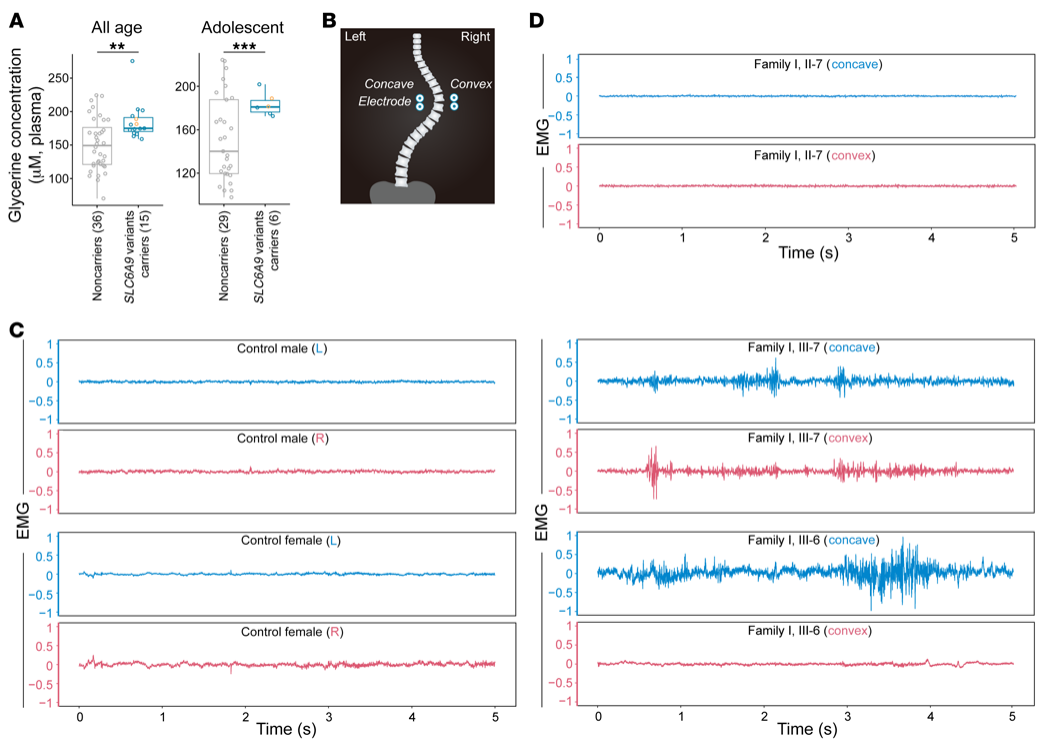
Plasma glycine concentration and paraspinal muscle activity in *SLC6A9* variant carriers. (**A**) Plasma glycine concentration was measured in 15 AIS patients carrying *SLC6A9* variants and 36 noncarrier controls (left panels). Plasma glycine concentration was measured in 6 adolescent patients carrying *SLC6A9* variants and 29 adolescent controls (right panel). Boxes show the median and IQRs, with all individual data points superimposed. Orange dots represent III-6 and III-7 in family 1. Unpaired Student’s *t* test. **P = 0.0012; ***P = 0.00080. (**B**) Placing positions of bipolar electrodes. Electrodes were positioned at thoracic vertebra T9-11 in controls or at apex vertebra in AIS patients. (**C**) sEMG signals from healthy controls (left) and preadolescent AIS patients (right). Raw sEMG signals were collected from the paraspinal muscle at thoracic vertebra T9-11 in controls or at apex vertebra (T9-11) in patients during 5 seconds of standing in an upright posture. Blue and red represent left (L) and right (R) or concave and convex sEMG signals, respectively. (**D**) sEMG signals from adult AIS patient II-7, father of III-6 and III-7, in family 1. Raw sEMG signals were collected from the paraspinal muscle at apex vertebra T5-7 during 5 seconds of standing in an upright posture.

Patients with glycine encephalopathy harboring homozygous *SLC6A9* mutations were reported to have increased glycine concentrations in the CSF or plasma (24, 25). Because the AIS patients in our study did not show any discernible neurological defects, it was not ethically justified to obtain CSF for measuring glycine concentrations. We instead measured plasma glycine concentration, which was found to be higher in AIS patients carrying *SLC6A9* variants (*n* = 15) compared with unaffected controls (*n* = 36). Moreover, if we only compared the individuals whose glycine levels were measured during adolescence, we identified a more significant difference between the *SLC6A9* variant carriers and noncarriers (Figure 2A). Notably, plasma glycine concentrations in 2 p.R662W variant carriers in family 1 (III-6 and III-7) were higher than in the controls, although these 2 subjects had yet to reach puberty and had no scoliotic phenotype at the beginning of the study. We followed these 2 high-risk children from age 6 to age 9 (III-6) and from age 9 to age 12 (III-7), respectively. They were both later diagnosed with mild spinal curvature (Cobb angle: 10° and 13.3°, respectively) from their latest spinal x-ray images (Supplemental Figure 2 and Supplemental Table 1), suggesting they were in the early stages of AIS development.

As glycine functions as a spinal cord neurotransmitter, we next measured the activity of the paraspinal muscles of AIS patients by surface electromyography (sEMG). The bipolar electrodes were positioned at the paraspinal muscles along the spine (Figure 2B). We examined the 2 aforementioned young patients in preadolescence (III-6 at age 9 and III-7 at age 12 in family 1) and 2 sex- and age-matched controls. The sEMG signals in the two affected children exhibited irregular bursts, indicative of the aberrant paraspinal muscle activity in AIS patients, whereas the controls showed stationary sEMG signals on both sides (Figure 2C). Interestingly, we failed to detect abnormal sEMG signals in an adult patient carrying the same *SLC6A9* variant (II-7, the father of III-6 and III-7 in family 1) (Figure 2D). These observations suggest that neuromuscular aberrations may be more pronounced if individuals are still in the initial stages of curvature development rather than a steady state after scoliosis has developed. The aberrant paraspinal muscle activity may adapt to the curvature progression in adult AIS patients and become coordinated with the well-established spinal curvature.

### Functional consequences of SLC6A9 variants on GLYT1.

**Figure 3:**
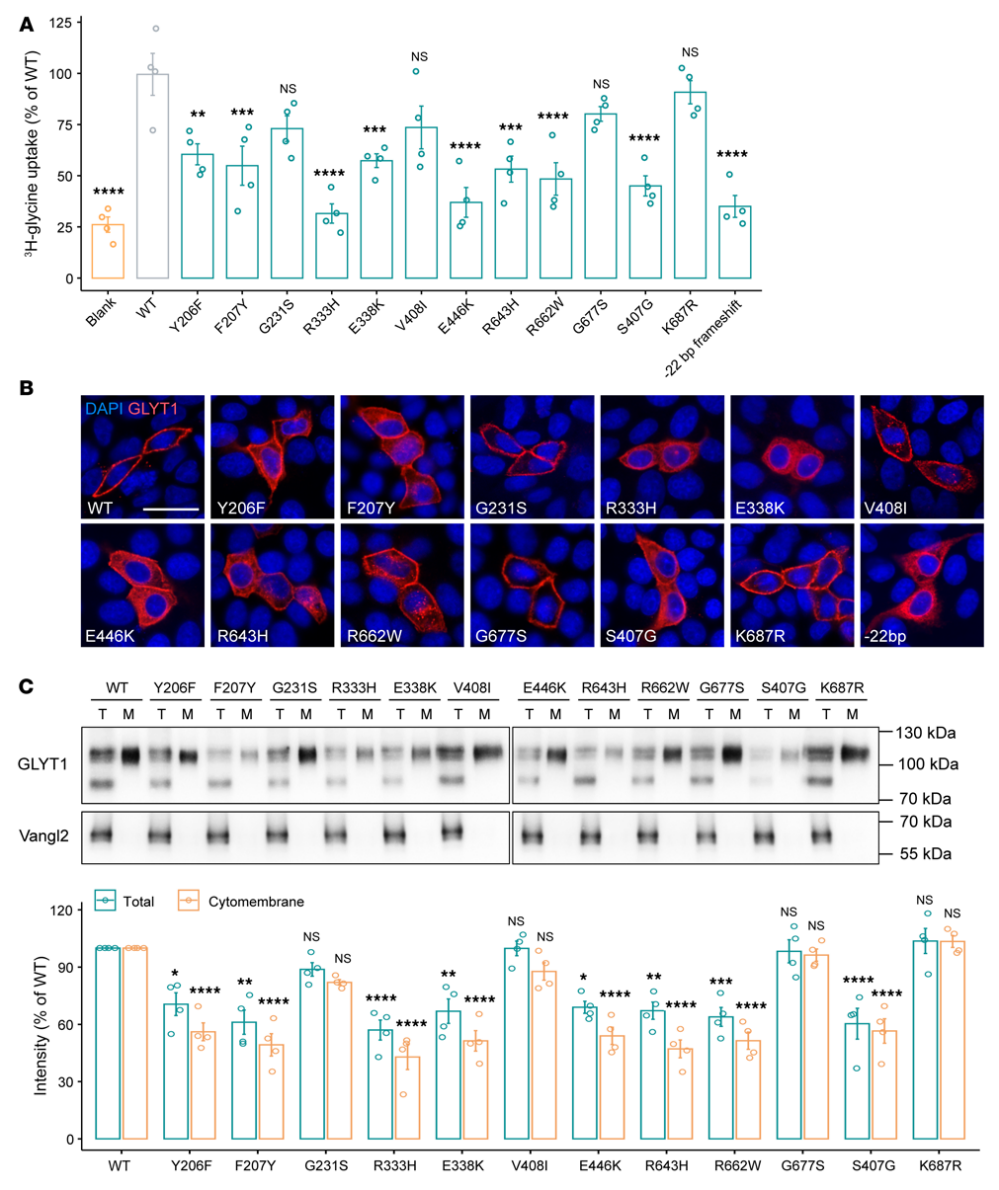
Effect of GLYT1 variants on glycine uptake and membrane presentation. (**A**) Results of glycine-uptake assay for GLYT1 in HEK293T cells. Each dot represents 1 independent experiment (*n* = 4). Error bars represent 95% CIs. Data are represented as means ± SEM. One-way ANOVA test. **P < 0.01; ***P < 0.001; ****P < 0.0001. (**B**) Subcellular localization of Flag-tagged GLYT1 in MDCK cells. Signals were visualized with anti-Flag antibody (red), and nuclei were stained with DAPI (blue). Scale bar: 20 μm. (**C**) Western blot analysis of Flag-tagged GLYT1 in total cell lysates (T) and biotinylated membrane fractions (M) of transfected HEK293T cells. Lower and higher bands indicate underglycosylated and glycosylated GLYT1, respectively. Expression of an unrelated membrane protein, HA-tagged Vangl2, served as internal transfection control. Quantification of immunoblots of total cell extracts and cytomembrane fractions of GLYT1 variants, normalized to Vangl2 and GLYT1 WT, is shown below. Each data dot represents 1 independent experiment (*n* = 4). Data are represented as means ± SEM. Two-way ANOVA test. *P < 0.05; **P < 0.01; ***P < 0.001; ****P < 0.0001.

Given the main function of GLYT1 in transporting extracellular glycine into cells, we assessed the glycine-uptake capacity of GLYT1 variants in HEK293T cells, which have no endogenous GLYT1 expression. K687R, a ubiquitination-deficient mutant that stabilizes GLYT1 on the cell surface (31), and S407G, a known mutation that causes recessive glycine encephalopathy (25), served as negative and positive controls, respectively. We found the [^3^H]-glycine uptake capacity of 7 variants (Y206F, F207Y, R333H, E338K, E446K, R643H, and R662W) was significantly reduced compared with that of WT GLYT1, whereas 3 other variants that have relatively higher allele frequency (G231S, V408I, and G677S) showed no significant differences (Figure 3A and Supplemental Table 3).

We next analyzed the subcellular localization of GLYT1 variants by immunofluorescent staining. WT GLYT1 was predominantly located on the cell surface, whereas GLYT1 variants (Y206F, F207Y, R333H, E338K, E446K, R643H, and R662W) were largely retained intracellularly. The localization of GLYT1 in cells expressing G231S, V408I, or G677S variants was minimally affected (Figure 3B and Supplemental Figure 4A). We further assessed the protein levels of GLYT1 variants, which showed all variants except G231S, V408I, and G677S had significantly lower levels of total and cytomembrane-associated GLYT1 (Figure 3C). After normalizing the glycine uptake activity of each variant with the corresponding cytomembrane protein level, we found no significant differences between the WT and variants (Supplemental Figure 4B), suggesting that decreased glycine-uptake activity might be a consequence of reduced levels of GLYT1 on the cell surface rather than due to the impairment of glycine-transporting function.

GLYT1 is a member of the Na^+^/Cl^–^-dependent neurotransmitter transporter (SLC6A) family, and these transporter family members can form dimers or oligomers (32). Several studies have shown that GLYT1 can form dimeric protein complexes not only in intracellular compartments but also in the plasma membrane (33–35). As all identified *SLC6A9* variants are heterozygous in AIS patients, it is possible that these variants also affect the function of WT GLYT1 in the complex. Therefore, we tested the effects of GLYT1 variants against WT GLYT1 by coexpressing them at a 1:1 ratio in cells. We found the majority of GLYT1 variants, including Y206F, F207Y, R333H, E338K, E446K, R643H, and R662W, impaired the localization and expression of WT GLYT1 (Supplemental Figure 5). Together, our results demonstrate that most of the identified *SLC6A9* variants from AIS patients (7 out of 10) caused loss of function and had dominant negative effects over WT GLYT1.

### Idiopathic scoliosis-like phenotype in slc6a9 mutant zebrafish.

Zebrafish have inherent advantages over other animal models and have been widely used for modeling AIS (36–38). We generated an *slc6a9* mutant zebrafish line, which carries a 22 bp deletion and produces a C-terminal truncated glyt1 (denoted as *slc6a9^m^* afterwards) (Supplemental Figure 6A). The cellular assays indicated that *slc6a9^m^* was a severe hypomorphic mutation. This mutant exhibited dominant negative effects over WT glyt1, recapitulating the characteristics of the *SLC6A9* missense variants identified in AIS patients (Figure 3, A and B, and Supplemental Figure 6, B–E).

**Figure 4:**
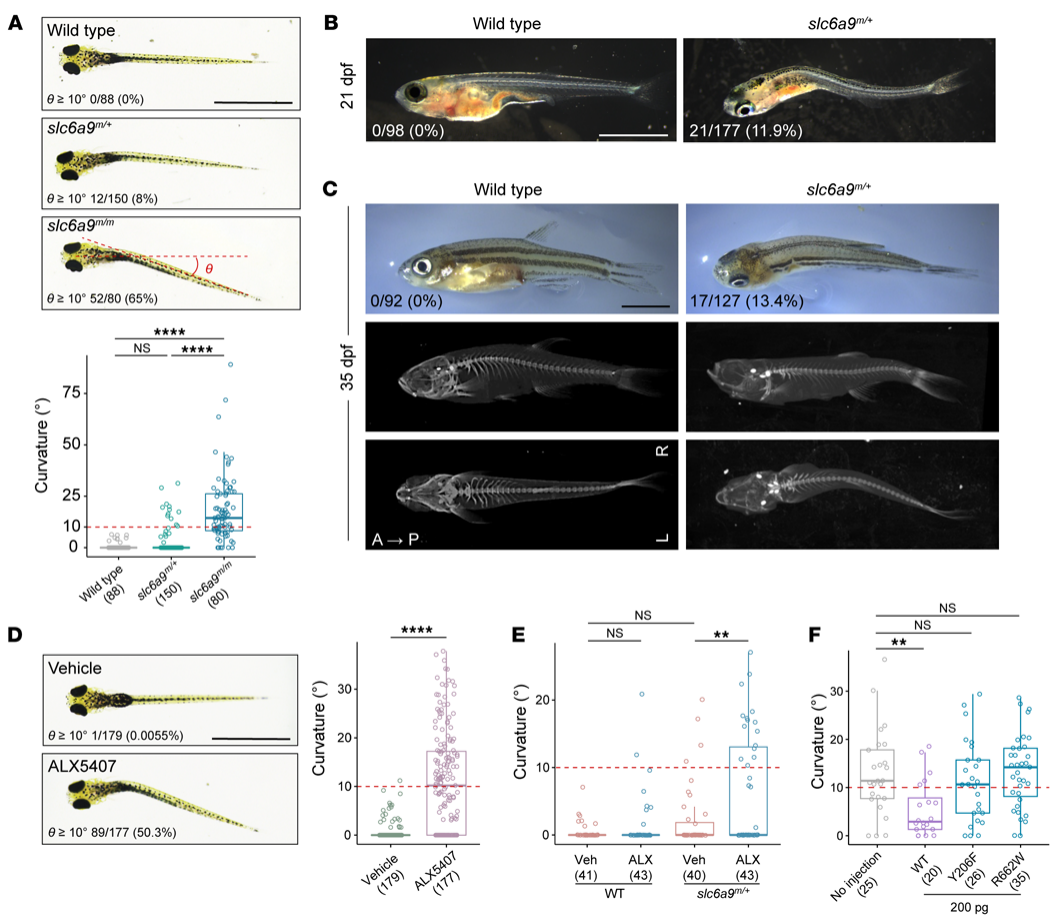
Body curvature in *slc6a9* mutant zebrafish. (**A**) Axial curvature of *slc6a9* mutant zebrafish at 7 dpf. The severity of the curvature is measured by θ angle. (**B**) Spinal curvature of *slc6a9* mutant zebrafish at 21 dpf. (**C**) Curvature phenotype and micro-CT images of WT and *slc6a9^m/+^* zebrafish at adolescent stage (35 dpf). Images are shown in either side or dorsal view. A, anterior; P, posterior; L, left; R, right. (**D**) Axial curvature of WT zebrafish larvae treated with vehicle or GLYT1 inhibitor ALX 5407 (1 μM). (**E**) Quantification of axial curvature in WT and *slc6a9^m/+^* zebrafish treated with vehicle (Veh) or low-dose ALX5407 (ALX, 10 nM). Only 10% of *slc6a9^m/+^* fish showed axial curvature (θ ≥10°), whereas 10 nM ALX5407 induced axial curvature in 4.65% of WT and 34.9% of *slc6a9^m/+^* fish. (**F**) Quantification of axial curvature in *slc6a9^m/m^* zebrafish with and without injection of 200 pg *SLC6A9* WT or mutant (Y206F or R662W) mRNAs. Scale bars: 1 mm (**A** and **D**); 2 mm (**B** and **C**). In all charts, boxes show the median and IQRs with all individual data points superimposed. The number of analyzed fish and the penetrance of curvature (θ ≥10°) are quantified and indicated for each genotype. Unpaired Student’s *t* test (**D**) or 1-way ANOVA test (**A**, **E**, and **F**). **P < 0.01; ****P < 0.0001.

Considering the crucial role of glyt1 in the survival of zebrafish (39, 40), we first assessed the survival rate of *slc6a9^m/+^* and *slc6a9^m/m^* mutant fish over time. The *slc6a9^m/m^* larvae began to die at 7 days post fertilization (dpf), with none surviving at 18 dpf. In contrast, around 50% of *slc6a9^m/+^* fish survived to the juvenile stage at 30 dpf (Supplemental Figure 7A). The extracellular glycine levels were significantly higher in *slc6a9^m/m^* fish compared with WT (Supplemental Figure 7B). The *slc6a9* mutants had no discernible morphological defects in motor neuron, skeletal muscle, axonal tract formation, and calcified vertebrae (Supplemental Figure 8 and Supplemental Figure 9A). Given the important role of notochord in zebrafish spine formation (38, 41), we examined the notochord sheath and the morphology of notochord vacuolated cells in *slc6a9^m/m^* mutant, which did not exhibit any anomalies in notochord (Supplemental Figure 9, B and C). Notably, at 7 dpf, 65% of *slc6a9^m/m^* fish showed an apparent lateral axial curvature (θ angle ≥10°) and approximately 8% of *slc6a9^m/+^* larvae exhibited a curvature phenotype (Figure 4A and Supplemental Videos 1–3). The locomotion of zebrafish, as measured by swimming distance, was significantly decreased in all *slc6a9* mutants (Supplemental Figure 10). By 18 dpf, we observed spinal curvature in all dying *slc6a9^m/m^* fish. As the *slc6a9^m/m^* fish did not survive beyond 18 dpf, we followed the phenotype of *slc6a9^m/+^* fish at later developmental stages. We found that 11.9% (21 of 177), 13.4% (17 of 127), and 12.5% (4 out of 32) of *slc6a9^m/+^* zebrafish exhibited overt body curvature at 21, 35, and 100 dpf, respectively. These zebrafish exhibited severe lateral spinal curvature and occasionally kyphosis, but showed no congenital vertebral malformation (Figure 4, B and C, and Supplemental Figure 11).

To further verify the causal effects of *slc6a9* on body curvature, we treated WT zebrafish larvae with ALX5407, a specific GLYT1 inhibitor (42). We observed an increase in curvature penetrance with the dose of ALX5407 (Supplemental Figure 12A), and 50.3% of WT fish treated with 1 μM ALX5407 showed obvious axial curvature and reduced free swimming distance, which is consistent with the phenotype of the *slc6a9^m/m^* mutant (Figure 4D and Supplemental Figure 12B). A low dose of 10 nM ALX5407 induced axial curvature in only 4.65% of WT fish, but significantly increased the penetrance (from 10% to 34.9%) and severity of curvature in the *slc6a9^m/+^* mutant (Figure 4E). Additionally, we characterized a reported *slc6a9* mutant line *ta229g*, which carries a G81D mutation causing disruption of transporter function (40). Intriguingly, 72.5% of *slc6a9^ta229g/ta229g^* mutant fish exhibited pronounced lateral curvature at 7 dpf, which has not been described in previous studies (Supplemental Figure 12C). Together, our results further support the importance of GLYT1 in the maintenance of spinal alignment in a dose-dependent manner.

To further investigate the functional consequence of the identified *SLC6A9* variants, we microinjected *SLC6A9* WT and AIS-associated variant mRNAs (Y206F and R662W) into the zygotes generated from *slc6a9^m/+^* and *slc6a9^m/+^* mating pairs. Notably, injection of 200 pg *SLC6A9* WT mRNA efficiently rescued the axial curvature of *slc6a9^m/m^* mutants (Supplemental Figure 13), whereas the same dosage of Y206F and R662W variant mRNAs failed to rescue the phenotype (Figure 4F). These results further demonstrate that the identified AIS-associated variants are indeed deleterious, accounting for the scoliosis phenotype.

### Scoliosis caused by dysfunction of CPGs in zebrafish.

**Figure 5:**
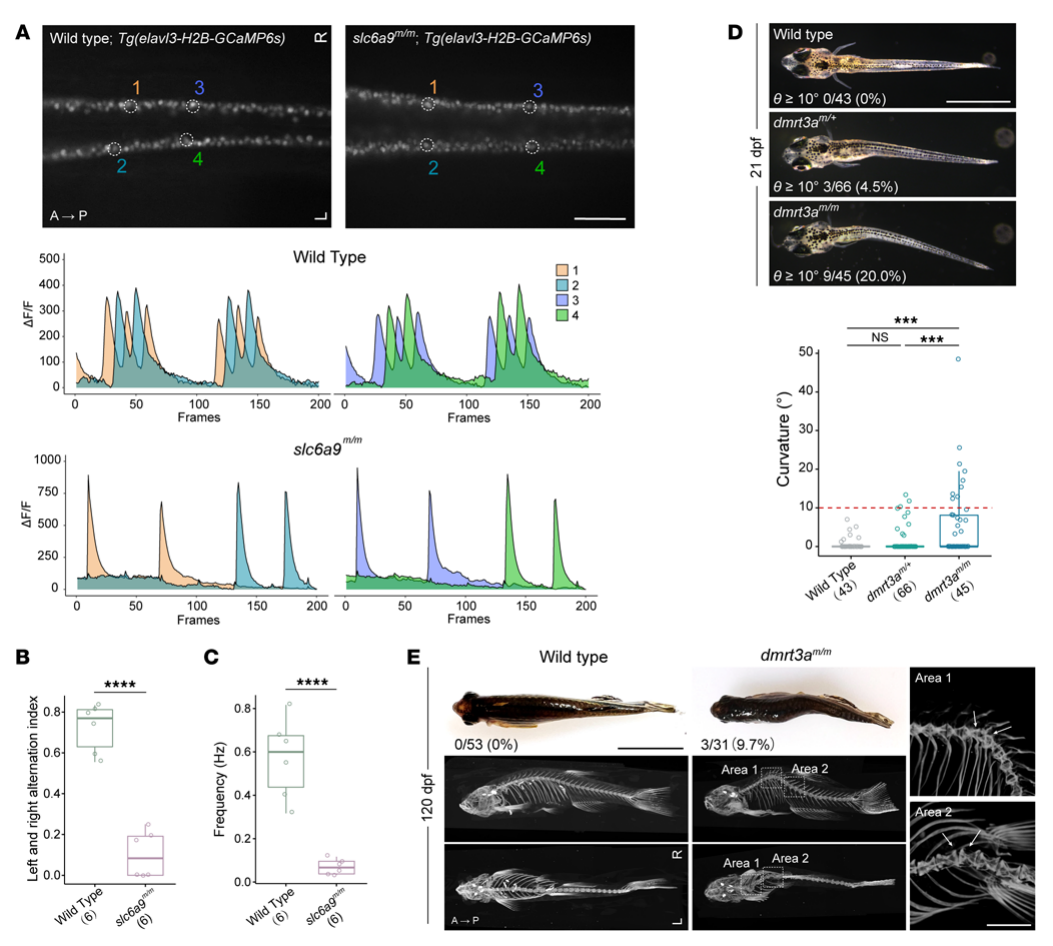
Body curvature caused by disturbance of CPG. (**A**) Dorsal view fluorescent snapshots of the spinal cord of WT and *slc6a9^m/m^* zebrafish in a *Tg(elavl3-H2B-GCaMP6s)* background at 24 hpf. ROI is circled and numbered as 1–4. Lower panel shows quantification of fluorescence changes in the ROIs of WT and *slc6a9^m/m^* zebrafish. Each frame was taken with a 100 ms exposure and at 10 fps. GCaMP6s fluorescence intensity was defined as the *ΔF/F*, and *ΔF/F* changes within a 20-second recording time are shown. (**B**) Quantification of left and right alternation index in WT (*n* = 6) and *slc6a9^m/m^* (*n* = 6) zebrafish. This analysis was performed based on quantified intensities of total left- and right-side neural activities within a 1-minute recording time period. Unpaired Student’s *t* test. ****P < 0.0001. (**C**) Frequency of neural activities in WT (*n* = 6) and *slc6a9^m/m^* (*n* = 6) zebrafish. Frequency (Hz) was calculated based on left-side neural activity. Unpaired Student’s *t* test. ****P < 0.0001. (**D**) Spinal curvature of *dmrt3a* mutant zebrafish at 21 dpf. (**E**) Curvature phenotype and micro-CT images of *dmrt3a* mutant zebrafish at 120 dpf. Images are shown in either side or dorsal view. To detect the details of apices of curvatures, the 2 curvature regions (areas 1 and 2) of *dmrt3a^m/m^* zebrafish are enlarged and oriented in different angles (right). Note that all highlighted adjacent vertebrae (arrows) are morphologically normal. Scale bars: 200 μm (**A**); 2 mm (**D**, **E**, right); 1 cm (**E**, left). Boxes show median and IQRs with all individual data points superimposed. Number of analyzed fish and the penetrance of curvature (θ ≥10°) are quantified and indicated for each genotype. Unpaired Student’s *t* test (**B** and **C**) or 1-way ANOVA test (**D**). ***P < 0.001; ****P < 0.0001.

CPGs are self-contained biological neural circuits that can generate tightly coupled patterns of neural activity driving rhythmic and stereotyped behaviors such as breathing, walking, and swimming independently of central commands or peripheral sensory inputs. The CPG involved in the control of locomotion is composed of spinal cord interneurons (43–47). As glycine is a major neurotransmitter in inhibitory interneurons of the spinal cord, excessive glycine caused by mutant GLYT1 may interfere with the normal function of CPGs. To understand the underlying mechanism that leads to the scoliosis-like phenotype in *slc6a9* mutant zebrafish, we next investigated their neural activity and found that a pattern of left-right alternation was disrupted in the *slc6a9^m/m^* spinal cord. The neural activities of WT fish are coordinated in both sides of the spinal cord or in specific pairs of neurons, as demonstrated by a calcium indicator in the *elavl3-H2B-GCaMP6s* transgenic background (48). However, such left-right alternating neural activity was impaired and abnormal unilateral neuronal activation was observed in the *slc6a9^m/m^* mutant (Figure 5A and Supplemental Figure 14A). The left-right alternation index was significantly decreased in the *slc6a9^m/m^* mutants, indicating that the total neuronal activities on one side are much stronger than on the other side (Figure 5B). Moreover, we observed that the signal frequency was significantly reduced in mutant fish (Figure 5C), which is consistent with the reduced swimming behaviors of *slc6a9^m/m^* mutant zebrafish. Notably, we also detected a significant total signal reduction in activated neurons in *slc6a9^m/m^* mutants, suggesting a strong inhibitory effect of the increased extracellular glycine (Supplemental Figure 14B).

To further test the role of CPGs in maintaining spinal alignment, we generated a *dmrt3a* mutant zebrafish line (denoted as *dmrt3a^m^* afterwards, Supplemental Figure 15A). Mutation in *DMRT3* or disruption of *Dmrt3/dmrt3a* is known to affect the pattern of locomotion. The *Dmrt3* mutant mice showed a significant decrease in commissural interneuron numbers and impaired limb coordination. The dI6 interneurons specified by transcription factor *Dmrt3* are important for proper left-right alternation of the body (49–51). We observed that *dmrt3a* mutant zebrafish exhibited reduced survival rates compared with WT (Supplemental Figure 15B). Interestingly, 10% to 20% of *dmrt3a^m/m^* zebrafish exhibited apparent lateral spinal curvature starting from approximately 18 to 21 dpf, with intact calcified vertebrae and no discernible congenital vertebral malformation (Figure 5, D and E, and Supplemental Figure 15C). These results further support the role of left-right coordination of rhythmic motor activities in maintaining spinal alignment. Taken together, our findings demonstrate that disturbance of CPGs by either excessive glycine or developmental defects can cause a scoliosis-like phenotype.

### Pharmacologic prevention of body curvature.

**Figure 6:**
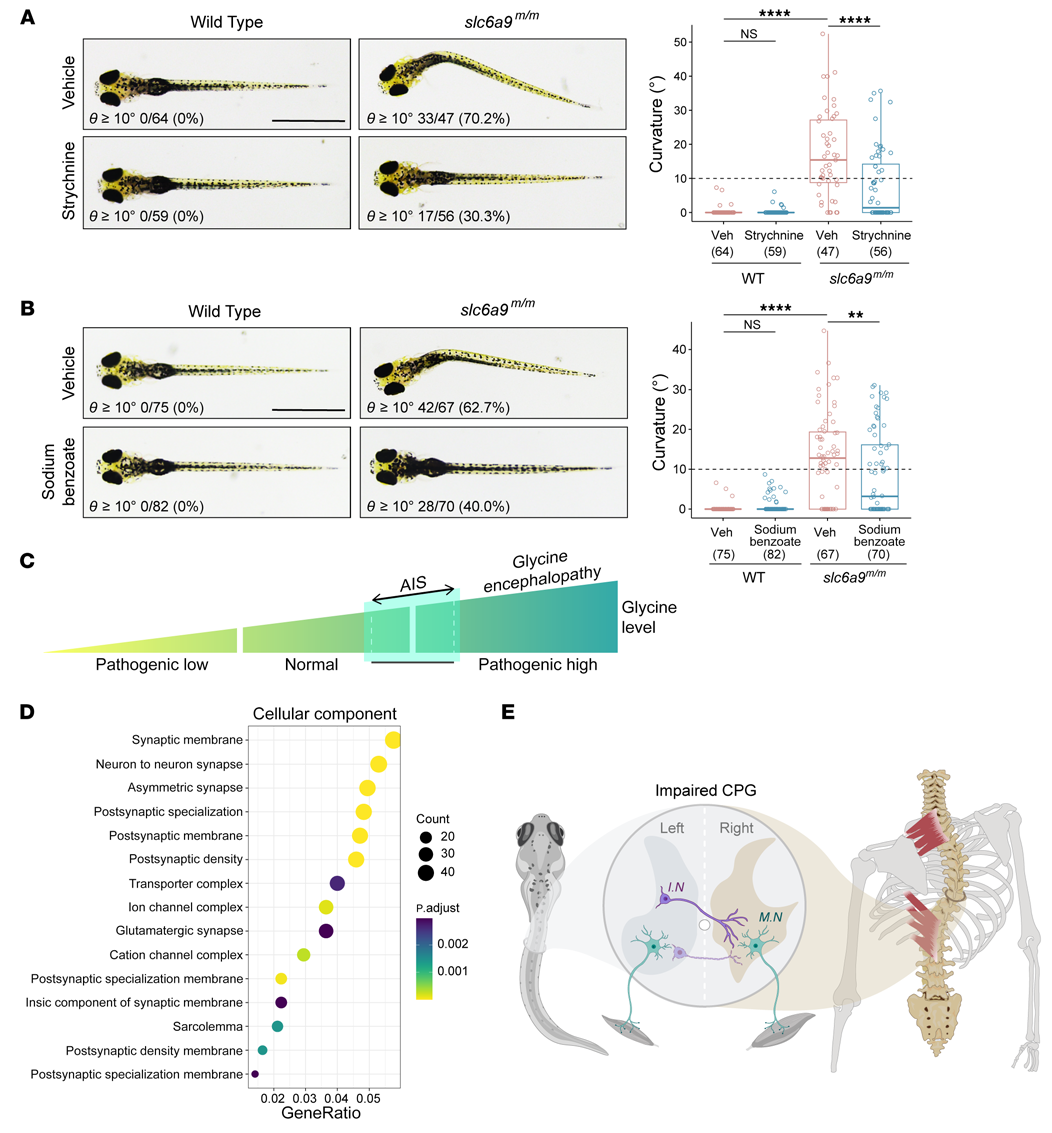
Prevention of body curvature in *slc6a9* mutant zebrafish by pharmacological intervention. (**A**) Representative dorsal-view images of axial phenotypes of WT and *slc6a9^m/m^* larvae treated with vehicle or strychnine (GlyR antagonist, 0.5 μM). (**B**) Representative dorsal-view images of axial phenotypes of WT and *slc6a9^m/m^* larvae treated with vehicle or sodium benzoate (glycine neutralizer, 0.5 ppm). Scale bars: 1 mm. In **A** and **B**, the number of analyzed fish and the penetrance of curvature are quantified and indicated for each genotype. Boxes show the median and IQRs with all individual data points superimposed. One-way ANOVA test. **P < 0.01; ****P < 0.0001. (**C**) Proposed glycinopathy spectrum. Abnormally high levels of glycine are associated with glycine encephalopathy, a severe neurological disease, whereas moderately elevated levels of glycine are a causal risk factor for AIS. (**D**) Cellular component of GO functional enrichment analysis of AIS-associated genes. GeneRatio is the ratio of genes mapped to a pathway to the total gene set. The size of the dots represents the number of genes mapped to the pathway. (**E**) Proposed disease mechanism of spinal curvature. In zebrafish, disruption of glyt1 causes a discoordination of left-right neural activities in the spinal cord due to aberrant glycinergic neurotransmission; deletion of *dmrt3a* partially impairs the development of commissural interneurons and compromises the locomotor left-right alternation; both lead to an AIS-like phenotype via the disturbance of CPGs in the spinal cord. In humans, functional impairment of GLYT1 leads to elevated glycine levels, aberrant paraspinal muscle activities, and AIS. Our findings suggest that dysfunction of the CPGs induced by either excessive glycine or developmental defects is one of the major causal factors underlying the etiology of AIS. I.N., interneurons; M.N., motoneurons.

Given that GlyRs are the main receptors on inhibitory postsynaptic terminals activated by synaptic glycine (52), we determined whether strychnine, a GlyR antagonist, could prevent the curvature phenotype of *slc6a9* mutant zebrafish (53). We found the strychnine treatment significantly reduced the number of *slc6a9^m/m^* mutants with axial curvature (θ angle ≥ 10°), from 70.2% to 30.3% (Figure 6A). We also attempted to block excessive extracellular glycine in mutant fish using sodium benzoate, which is a glycine neutralizer that is clinically used to treat patients with glycine encephalopathy (54). We observed a moderate decrease in the number of *slc6a9^m/m^* mutants showing a curvature phenotype from 62.7% to 40.0% (Figure 6B). Both strychnine and sodium benzoate treatments markedly reduced the severity of curvature (Figure 6, A and B), which suggests that neutralizing or blocking the activity of excessive glycine in the mutant zebrafish can partially rescue the idiopathic scoliosis-like phenotype.

## Discussion

Our work explores the genetic basis and pathogenic mechanisms that lead to the development of idiopathic scoliosis in adolescents. By inheritance mapping in 2 large families with dominant inheritance of AIS, we identified an AIS locus on chromosome 1p34.1 and the causal gene *SLC6A9*. We further identified a number of missense *SLC6A9* variants in diverse AIS cohorts. The extremely low frequency or lack of p.R662W variant in global or local populations supports its causal effect in family 1. Other variants identified in sporadic cases are also extremely rare in the general population. Interestingly, the p.Y206F variant was prevalent in the Hong Kong AIS cohort, presenting in 1 large family, 3 trios, and 11 sporadic patients, which accounts for 1.769% (15 of 848) of AIS cases studied in Hong Kong. The presence of the p.Y206F variant in the general populations (0.029% to 0.560%) may reflect its relatively low penetrance in causing AIS and/or a lack of diagnosis for mild scoliosis in the control groups. Our functional assays provide evidence that the majority of the identified *SLC6A9* variants caused markedly decreased protein levels and impaired cell-surface presentation of GLYT1, consequently resulting in decreased glycine uptake. As complex membrane proteins are often subjected to quality control mechanisms in the endoplasmic reticulum (ER) (55), mutant GLYT1 may be degraded by proteasome via the ER-associated degradation (ERAD) pathway or be unstable on the cell surface and prone to endocytosis and lysosomal degradation. Our studies in animal models further showed that *slc6a9* mutant zebrafish exhibited spinal curvature in an *slc6a9* gene and GLYT1 inhibitor dose-dependent manner. These genetic and mechanistic studies collectively demonstrate a functional role of *SLC6A9* in AIS etiology.

Despite extensive studies on idiopathic scoliosis, the etiopathogenesis of AIS has remained obscure and controversial. The high incidence of scoliosis in children with neurological diseases (neuromuscular scoliosis) has led to the neuropathic hypothesis for AIS, in which a small scoliotic curve may initially develop due to a small defect in the nervous system, either from altered sensory input or altered neuromuscular control. This defect produces asymmetric muscle loading or loss of muscle support that directly leads to the initiation of the scoliotic deformity, which is further exacerbated by biomechanical factors during the adolescent growth spurt. Therefore, it has long been proposed that one of the likely causes of AIS is neuromuscular and that AIS may be a late-onset subtype or mild form of neuromuscular scoliosis (56). Because such neurological defects are subtle, not detectable by conventional clinical assessment, patients were diagnosed with “idiopathic” rather than neuromuscular scoliosis. However, convincing evidence for this hypothesis, especially human genetic evidence with mechanistic proof for the causal relationship, was largely lacking. Studies in mouse models showed that defects in the proprioceptive system caused by deletion of *Runx3* or ablation of mechanosensor Piezo2 resulted in spinal misalignment (57, 58). Genetic studies in zebrafish indicated that abnormalities in cilia or cilia-mediated CSF flow, dysfunction of Reissner’s fiber, or activation of proinflammatory signals within the spinal cord were associated with idiopathic-like scoliosis (36, 59–65). Previous studies reported null variants of *PIEZO2* in 3 families and 2 patients with neuromuscular symptoms and progressive scoliosis, indicating a connection between mechanosensory defects and development of spinal curvature (66, 67). In the past decade, population genetic studies have identified many susceptibility genes for AIS, and the most significant one is *LBX1* (13, 14, 18, 19, 68–70). *LBX1* plays critical roles in specifying dorsal spinal neurons and hindbrain somatosensory neurons, suggesting a potential etiology through abnormal neural function (71, 72). Here, the identification of *SLC6A9* variants in many familial and sporadic cases extends the spectrum of glycinopathy manifestation and implies a role of glycine synaptic transmission in the etiology of AIS. A moderate elevation of glycine may be a strong risk factor for AIS (Figure 6C). Intriguingly, a functional enrichment analysis of all reported AIS susceptibility variants revealed the vast majority of the enriched pathways are associated with synaptic homeostasis (Figure 6D and Supplemental Figure 16). These findings strongly suggest a neuropathic origin of AIS in a considerable proportion of patients.

The neuropathic hypothesis has led to repeated attempts to identify a neuromuscular cause of AIS. One of the efforts involves measuring and comparing sEMG activities of the paraspinal muscles in patients and controls. Previous work mainly studied patients aged 12 to 19 years, which identified either an increased myoelectric response on the convex or concave side of the scoliotic curve or no differences between sides (73). It is unclear whether such change is the primary cause or a secondary phenomenon induced by the deformed spine. In this study, we found that healthy controls had stationary sEMG signals, but 2 children aged 9 and 12 carrying the *SLC6A9* pathogenic variant showed aberrant sEMG bursts, which may reflect an impairment of the balance of the paraspinal muscle control at the preadolescent stage. These findings are consistent with the left-right coordination defects in *slc6a9* mutant zebrafish, while zebrafish studies provide more accurate and much higher resolution of neural activities than human sEMG. Considering the normal sEMG in an adult patient carrying the same pathogenic *SLC6A9* variant and the conflicting sEMG in relatively mature AIS patients (73, 74), we argue that spinal curvature is a compensatory response that eventually corrects or adapts to the aberrant paraspinal muscle activity in older patients and therefore the findings from mature AIS patients varied greatly (73). Measuring sEMG on the paraspinal muscle of asymptomatic or early stage patients would be more informative. Our results suggest that disturbance of bilateral paraspinal muscle control might be a causal factor for AIS, which warrants further large-scale prospective studies in the preadolescent population (e.g., ages 8 to 12). sEMG screening in preadolescent children and long-term follow-up may allow us to determine whether aberrant paraspinal muscle activities can be used as a biomarker for identifying potential AIS patients for preventive treatment.

Distinct CPGs located throughout the CNS mediate various biological rhythms (44). Left-right alternation of locomotion is thought to be organized by glycinergic commissural inhibitory neurons. Previous studies showed that the V0 commissural interneurons play a fundamental role in securing left-right alternation in the locomotor CPG (43, 45, 46, 75). In particular, the inhibitory V0 neurons are required for left-right alternating modes during slow locomotion (75). Given the indispensable function of GLYT1 in inhibitory glycinergic neurotransmission and the defects of left-right alternation observed in the spinal cord of *slc6a9* mutant zebrafish, we speculate that excessive synaptic glycine may compromise the function of CPGs, leading to an imbalance in neuromuscular activity of the paraspinal muscles and thus loss of spinal alignment (Figure 6E). Besides, the Dmrt3^+^ dI6 commissural interneurons are also reported to function in left-right alternation (51). The AIS-like phenotype induced by loss of *dmrt3a* in zebrafish further supports the functional role of CPGs in spinal alignment. The overall penetrance of spinal curvature in *dmrt3a* mutants was lower and onset was later than in *slc6a9* mutants. This is likely due to compensatory effects of WT1^+^ dI6 interneurons (49). Notably, Dmrt3^+^ interneurons and WT1^+^ interneurons are both derived from Lbx1^+^ lineage of interneurons in the dorsal spinal cord (49, 71, 72). As *LBX1* is so far the most important predisposing gene replicated in multiethnic populations for AIS, this further implicates a critical role of CPGs in causing AIS. Moreover, contralateral projection of the commissural axon is required for the development of CPGs (45). Several genes encoding commissural axon guidance molecules, including *ROBO3*, *EPHA4*, *CHL1*, and *DSCAM*, are strongly associated with scoliosis (18, 76, 77). Mutations in *ROBO3* cause horizontal gaze palsy with progressive scoliosis (HGPPS), which is a rare disorder that affects the spine and vision (77). EphA4-positive neurons constitute a critical component of locomotor CPG (78). This evidence implies that CPG dysfunction may be a common causative factor in the development of AIS.

The markedly reduced left-right alternation index in *slc6a9* mutant zebrafish indicates that the total level of neuronal activities on one side is much greater than on the other, suggesting that the contractions of muscles on one side are much greater than on the other. This imbalance of bilateral muscle contractions can initiate axial curvature. The mutant fish exhibited axial curvature to varied extents, and the persistent unbalanced paraspinal muscle activities continued to bend the body and make it curved most of time (Supplemental Videos 1–3). However, how such curvature became fixed or permanent at the late stages is an unresolved question. It is possible that the initial curvature is exacerbated by other mechanisms, such as biomechanical factors or secondary bony structural changes during the adolescent growth spurt, that ultimately misshape the spine. This warrants more investigation in the future.

Glycine has many potential health benefits, such as improving sleep, elevating mood, and lowering the risk of heart disease (79–81). Glycine is found in many protein-based food sources and is used as a food additive or taken as supplements. However, the long-term safety of glycine supplements, such as their effects on plasma or CSF glycine levels, has not been fully tested. We observed an increased penetrance and severity of axial curvature in *slc6a9^m/+^* zebrafish after administering a glyt1 antagonist, ALX5407, which specifically elevates glycine levels in the CSF (24). Our work raises the possibility that high levels of CSF glycine increase the risk of developing AIS in children and adolescents, especially in those who carry genetic susceptibility variants. It would be highly valuable to investigate whether there are associations among glycine levels, sEMG signals, and genetic variants with AIS, which could establish a new method for predicting AIS in preadolescents. Our data also suggest that pharmacologic interventions may be considered for preventing or alleviating the scoliotic phenotype in humans. Both strychnine and sodium benzoate treatments markedly reduced the severity of curvature in zebrafish. Although strychnine is highly toxic to humans (82), sodium benzoate is recognized as safe by the FDA and used as a treatment for a variety of human diseases, including urea cycle disorders, schizophrenia, and glycine encephalopathy (54). The moderate rescue of curvature in the animal model suggests sodium benzoate could be a potential preventive therapy in AIS patients with high levels of glycine. Our work lays the foundation for further investigations on the etiopathogenesis, early diagnosis, and potential pharmacological interventions for AIS.

## Methods

### Study participants.

Subjects diagnosed with AIS were recruited from the DKCH, PUMCH, and SRC. Diagnosis was made by standing whole-spine radiographs. Eligible subjects were patients diagnosed with scoliosis (Cobb angle ≥10°) without manifestation of any congenital or neuromuscular defect at the time of recruitment (Supplemental Figure 2 and Supplemental Table 1). Bilateral sEMG was recorded in a sample group of patients in comparison with 2 controls matched by sex and age. We recruited five 3-generation pedigrees from DKCH. We performed WGS on all families and further evaluated 2 families, families 1 and 2, with an autosomal dominant inheritance pattern because they shared a common candidate gene. The probands of family 1 and family 2 were initially identified at the time of their scoliosis surgery. Further genealogical investigation led to the identification of 2 large multiplex families (Figure 1A). We also consecutively recruited a total of 843 sporadic AIS cases, of which 118 patients were subjected to whole-exome sequencing (WES) and 725 patients were analyzed by targeted sequencing. Further genealogical and genetic investigations of the sporadic patients identified 3 trio families with apparent dominant inheritance (family 3-5) (Figure 1, A–C). We also recruited 3,219 ethnicity-matched subjects in Hong Kong with no evidence of AIS confirmed by radiographs as the general population controls. We further enrolled 2 additional AIS cohorts from PUMCH (*n* = 223) and SRC (*n* = 635) (Figure 1C). Among the cases from PUMCH, 120 and 103 individuals were analyzed by WES and WGS, respectively. All cases from SRC were analyzed by WES.

### Plasma glycine concentration assay.

The following *SLC6A9* variant-carrying AIS patients were recruited for measuring plasma glycine concentrations: 9 patients (II-1, II-3, II-5, II-7, II-8, III-2, III-3, III-6, and III-7) from family 1; 2 patients (II-7 and II-9) from family 2; I-2 from family 3; I-1 from family 5; and 2 sporadic patients (PUMCH_1 and PUMCH_2) from PUMCH. The unaffected family members without *SLC6A9* variants were recruited as a control group, including 4 members (I-1, II-2, II-6, and III-1) from family 1, 3 members (II-5, II-10, and III-1) from family 2, and I-1 from family 3. An additional group of 28 adolescents without AIS were recruited and served as age-matched general controls. Fasting plasma samples of recruited individuals were isolated from fresh whole blood by centrifugation at 4000 rpm for 15 minutes. The glycine concentration was measured according to the manual of the fluorometric glycine assay kit (Abcam, ab211100). Fluorescence was read at Ex/Em 535/587 nm in endpoint mode using a microplate reader (Varioskan Flash, Thermo Fisher Scientific).

### sEMG.

sEMG signals were detected with an amplifier of 1,000 times, sample frequency of 2,000 Hz, and filtering band of 15 to 1,000 Hz (YRKJ-A2004, Zhuhai Yiruikeji Co.). Back skin of participants was cleansed with 75% alcohol before electrode placement. Four pairs of silver/silver chloride self-adhesive surface electrodes (Noraxon Dual Electrode) were applied on bilateral paraspinal muscles at thoracic vertebra levels of T3-5 and T9-11 in III-6 and III-7 from family 1 and T3-5 and T5-7 in II-7 from family 1. Signals from T3-5 were used to remove ECG contamination (83). The recording at T9-11 or T5-7 reflected the paraspinal muscles at the apex of the spinal curvature as illustrated (Figure 2B). All subjects were asked to lie on a test bed for surface electrode placement, while the impedance was tested under 10 kΩ. Then they were instructed to relax and stand in an upright posture for 5 seconds as well as proceed with left and right trunk bending. The sEMG signals during left and right trunk bending were used as normalization of standing sEMG measurements. Raw sEMG signals were preprocessed with filtering, zero mean, and ECG removal.

### In vitro glycine uptake assay.

HEK293T cells were plated onto poly-l-lysine–coated 24-well plates (Sigma-Aldrich, P6282) and grown to 50%-60% confluence. Cells were transfected with Flag-GLYT1 WT, Flag-GLYT1 variants, and pCMV-3Tag-1A backbone. The detailed procedure of glycine uptake assay was described previously (84). Briefly, prior to uptake, the cells were washed 3 times with assay buffer containing 116 mM NaCl, 1 mM NaH_2_PO_4_, 26 mM NaHCO_3_, 1.5 mM MgSO_4_, 5 mM KCl, 1.3 mM CaCl_2_, and 5 mM glucose, and then incubated for 10 minutes with 1 μCi/mL [^3^H] glycine (60 Ci/mmol, PerkinElmer, NET004001MC) at a final concentration of 200 μM at 37°C. Glycine uptake was terminated by quick washing with ice-cold assay buffer followed by aspiration twice. Cells were digested in 0.1M NaOH, and the supernatants were subjected to scintillation counting (LS6500, Beckman Coulter) and protein concentration measurement using Bradford reagent (Pierce Coomassie Plus Assay Kit, Thermo Fisher Scientific, 23236). [^3^H]-glycine uptake was calculated as nanomoles per minute per milligram of protein (nmol/min/mg protein) and normalized as a percentage of that in control cells transfected with WT plasmid.

### Zebrafish lines.

Zebrafish embryos were collected from natural mating, maintained in E3 medium at 28.5°C, and staged according to dpf and morphology (85). WT zebrafish (TU) were used to generate the *slc6a9* mutant, *dmrt3a* mutant, and *Tg(elavl3-H2B-GCaMP6s)* transgenic zebrafish lines. The *slc6a9* mutant zebrafish were crossed into the *Tg(mnx1:GFP)* background to visualize the motoneurons or into the *Tg(elavl3-H2B-GCaMP6s)* background to measure neural activities (48, 86). The zebrafish *slc6a9* *ta229g* line has been described previously and was obtained from the National BioResource Project Zebrafish (Japan) (40).

Zebrafish genome and transcript information were derived from the updated zebrafish genome annotation (GRCZ11) in the *Ensembl* database. CHOPCHOP (https://chopchop.cbu.uib.no/) and CRISPRscan (http://www.crisprscan.org/) were used to design sgRNAs (87, 88). The sgRNAs targeting the last exon of *slc6a9* (CCGTGGCGTATCGACCCTTG) and the first exon of *dmr3a* (TGCGCGCTGCAGGAACCACG) with minimal off-targeting scores were, respectively, selected. A Nikon SMZ 745T stereomicroscope with Warner Pico-Liter Injector PLI-90A and 3D Manual Micro-Manipulator Fits Micropipette with OD 1 mm platform was used for microinjection, and 1 nL drop of a mix of 100 ng/μL Cas9 mRNA (Alt-R S.p. Cas9 Nuclease V3, Integrated DNA Technologies, 1081059) and 300 ng/μL synthesized sgRNA (Synthego) were microinjected into WT zygotes at the 1-cell stage. The mutant zebrafish lines carrying a deletion of 22 bp mutation of *slc6a9* and a deletion of 8 bp mutation of *dmrt3a* were established, respectively. The founder was bred to WT zebrafish to generate F0 and subsequent F1 mutants. The *slc6a9* allele was genotyped using the following primers: *slc6a9*-F: AGCACAGCAACTTTTCCAACC; *slc6a9*-R: TGCTTCCTGGGATGGTCAGA. The PCR product sizes of WT and mutant *slc6a9* allele were 255 and 233 bp, respectively. For genotyping the *dmrt3a* WT allele, *dmrt3a*-WT-F: CTGCAGGAACCACGGGGT and *dmrt3a*-R: AAGTTGCCAGTGTCAATGTT were used. For genotyping the *dmrt3a* mutant allele, *dmrt3a*-M-F: GCGCGCTGCAGGGGTGCTGT and *dmrt3a*-R were used. The PCR product sizes of WT and mutant *dmrt3a* allele were 501 bp and 498 bp, respectively.

The *Tg(mnx1:GFP)* zebrafish line that labels motoneurons has been described previously and was obtained from YSY Biotech (86). To visualize neuronal calcium signals in *slc6a9* mutant larvae, we generated a transgenic zebrafish line, *Tg(elavl3-H2B-GCaMP6s)*, using Tol2 construct with elavl3 promoter and human histone H2B that drive the expression of calcium indicator GCaMP6s in the nucleus of all neurons (48). The Tol2-elavl3-H2B-GCaMP6s construct was microinjected with *Tol2* mRNA into zebrafish zygotes at the 1-cell stage. The founder fish were crossed with the WT fish to establish the *Tg(elavl3-H2B-GCaMP6s)* line, which was further mated with *slc6a9* mutant fish to generate *slc6a9^m/m^;Tg(elavl3-H2B-GCaMP6s)*.

### Analysis of spinal neural activity.

Spinal neural activity of *Tg (elavl3-H2B-GCaMP6s)* and *slc6a9^m/m^*;*Tg (elavl3-H2B-GCaMP6s)* fish was analyzed at 24 hpf. Fertilized eggs were immobilized by 100 μM d-tubocurarine chloride hydrate (Abcam, ab120073) in E3 medium for 10 minutes and embedded with 0.8% to 1% low-melting agarose gel in the lid of 6 cm confocal dishes with desired direction. Neural activity reflected by GCaMP6s calcium signals was visualized by a Nikon Ti2-E Widefield Microscope with a ×40 air LEN. Signals were recorded continuously in a single *z* plane, and a time series mode was used to record the changes of neuronal GCaMP6 signals within 1 minute at a speed of 10 frames per second (fps). The recorded images were analyzed by ImageJ software (NIH). The ImageJ Time Series Analyzer plugin was used to manually quantify GCaMP6s signals. To characterize calcium signals of specific regions, the regions of interest (ROIs) were defined and the GCaMP6s fluorescence intensities (*ΔF*) of the time-lapse images of each fish were automatically extracted. *ΔF* for ROI was calculated as *ΔF* = *F*(*t*) − *F*0, where *F*0 is a manually selected baseline and *F*(*t*) is the GCaMP6s fluorescence intensity at a given time. Relative intensity of GCaMP6s signals was normalized as a percentage of the mean value of *ΔF*. The left and right alternation index was defined as the number of consecutive pairs of patterned events occurring on opposite sides of the spinal cord, divided by the total number of events minus 1. To compare the alternation index in WT and mutant zebrafish, quantified intensities of total left- and right-side neuronal activities within a 1-minute recording time period were used. Frequency of the left-side neuronal activity was quantified as Hz.

### Micro-CT.

Experimental zebrafish were euthanized with overdosage of MS222 solution (>250 mg/L, Sigma-Aldrich, 10521) and were fixed in 10% neutral-buffered formalin (Sigma-Aldrich, HT501128) overnight at 4°C. Fish were secured in the micro-CT instrument (Skyscan 1076, Burker). The parameters that we used for micro-CT scanning were as follows: source voltage, 40 kV; source current, 250 μA; pixel size, 8.6650 μM without filter.

### Drug treatment.

To phenocopy the axial curvature observed in 7 dpf *slc6a9* mutant zebrafish, a selective GLYT1 inhibitor ALX5407 (Sigma-Aldrich, SML0897) was used to treat the WT larvae (42). At 48 hpf, WT embryos were divided into a vehicle group (kept in E3 medium) and multiple treatment groups, in which the embryos were transferred to fresh E3 medium containing different dosages of ALX5407. Culture medium was changed daily. At 7 dpf, fish from vehicle and ALX5407 treatment groups were imaged for axial phenotype and tracked for swimming behaviors for over 10 minutes.

To enhance the penetrance of axial curvature observed in 7 dpf *slc6a9^m/+^* zebrafish, low dosage of ALX5407 was used. At 48 hpf, embryos from WT and *slc6a9^m/+^* mating pairs were divided into a vehicle group (E3 medium) and a treatment group (E3 medium containing 10 nM of ALX5407). At 7 dpf, fish from vehicle and treatment groups were imaged for axial phenotype and then lysed for genotyping.

A specific GlyR antagonist strychnine (Sigma-Aldrich, S0532) was used to prevent the axial curvature observed in 7 dpf *slc6a9^m/m^* zebrafish. The 6 dpf fish from *slc6a9^m/+^* and *slc6a9^m/+^* mating pairs were divided into vehicle group and treatment group. In vehicle group, fish were kept in E3 medium, whereas in treatment group, fish were kept in E3 medium containing 0.5 μM strychnine. After 24 hours, all fish were imaged for axial phenotype and then lysed for genotyping.

The human body can rapidly clear sodium benzoate by combining it with glycine to form hippuric acid for excretion (54). Hence, sodium benzoate (Sigma-Aldrich, B3420) was used as a neutralizer for glycine molecules in zebrafish to determine whether it can prevent the axial curvature of *slc6a9^m/m^* zebrafish. The 2 dpf embryos from *slc6a9^m/+^* and *slc6a9^m/+^* mating pairs were divided into a vehicle group and a treatment group. In the vehicle group, larvae were kept in E3 medium, whereas the larvae from the treatment group were kept in E3 medium containing 0.5 ppm sodium benzoate. Culture medium was changed daily. At 7 dpf, all fish were imaged for axial phenotype and then lysed for genotyping.

### Functional enrichment analysis of AIS GWAS data set.

A total of 1,387 SNPs that were significantly associated with AIS and mapped to 1,367 genes were collected from the NHGRI-EBI GWAS catalog database (89). Gene Ontology (GO) function enrichment analysis of these AIS-associated genes was performed by the clusterProfiler R package (90).

Additional methodological information is provided in the Supplemental Methods.

### Statistics.

Statistical data were analyzed using GraphPad Prism 7 (GraphPad Software). Student’s *t* test or 1-way or 2-way ANOVA was performed accordingly as indicated in the figure legends. Differences with *P* values of less than 0.05 were considered statistically significant. The *n* numbers for each group and group numbers are indicated in the figure or figure legends.

### Study approval.

Ethics approvals were obtained from the Institutional Review Board of the University of Hong Kong/Hospital Authority Hong Kong West Cluster (HKU/HA HKW IRB, reference UW 08-158), PUMCH under the framework of the Deciphering Disorders Involving Scoliosis and COmorbidities (DISCO) study (JS-3545D), and the Institutional Review Board of the University of Texas Southwestern Medical Center (STU 112010-150), respectively. Written, informed consent was obtained from all participants and the participating family members. Zebrafish experiments were conducted in compliance with the Guidelines from The Committee on Use of Laboratory Animals for Teaching and Research (CULATR) of the University of Hong Kong (CULATR 5396-20).

### Data availability.

Values for all data points in graphs are reported in the Supporting Data Values file. Large-size raw genotyping and sequencing data are available for access upon reasonable request.

## Author contributions

JPYC, YQS, and BG conceived the project. JPYC, PWHC, JJR, WT, GQ, ZW, TJZ, SI, NW, CAW, and KDKL recruited patients. Xiaolu Wang, MY, JPYC, and PWHC curated data. Xiaolu Wang, MY, YF, MW, Xiaojun Wang, SZ, AMK, ZC, Xiwei Wang, and YH developed methodology. Xiaolu Wang, MY, YF, MW, Xiaojun Wang, SZ, AMK, and JJR performed experiments. Xiaolu Wang and MY visualized experiments. JPYC, QY, DQ, GQ, ZW, TJZ, SI, NW, CAW, YQS, and BG acquired funding. JPYC, YQS, and BG performed project administration. JPYC, WT, DC, SI, NW, CAW, YH, YQS, and BG supervised the project. Xiaolu Wang, MY, JPYC, YQS, and BG wrote the original draft. Xiaolu Wang, MY, JPYC, SZ, AMK, DC, SI, NW, CAW, YH, KDKL, YQS, and BG reviewed and edited the manuscript. Xiaolu Wang, MY, and JPYC share the first author position in the given sequence for their specific contributions based on workload and significance to the project.

## Supplementary Material

Supplemental data

Supplemental tables 1-5

Supplemental video 1

Supplemental video 2

Supplemental video 3

Supporting data values

## Version 1 11/14/2023

In-Press Preview

## Version 2 01/16/2024

Electronic publication
