# Supplementary material for: Impaired glycine neurotransmission causes adolescent idiopathic scoliosis
Source: J Clin Invest. 2024 Jan 16;134(2):e168783. doi: 10.1172/JCI168783 (PMC10786698; doi:10.1172/JCI168783)
Supplement: Supplemental data [file jci-134-168783-s188.pdf]

Supplementary Materials for

Impaired Glycine Neurotransmission Causes Adolescent Idiopathic Scoliosis

Xiaolu Wang, Ming Yue, Jason Pui Yin Cheung, Prudence Wing Hang Cheung, Yanhui Fan, Meicheng Wu, Xiaojun Wang, Sen Zhao, Anas M Khanshour, Jonathan Rios, Zheyi Chen, Xiwei Wang, Wenwei Tu, Danny Chan, Qiuju Yuan, Dajiang Qin, Guixing Qiu, Zhihong Wu, Terry Jianguo Zhang, Shiro Ikegawa, Nan Wu, Carol A. Wise, Yong Hu, Keith Dip Kei Luk, You-Qiang Song, Bo Gao.

Correspondence to:
Dr. Jason Pui Yin Cheung (cheungjp@hku.hk)
Dr. You-Qiang Song (songy@hku.hk)
Dr. Bo Gao (bogao@cuhk.edu.hk)

This PDF file includes:

Supplementary Methods
Supplemental Figs. 1 to 16
Captions for Supplemental Tables 1 to 5
Captions for Supplemental Movies 1 to 3

Other Supplementary Materials for this manuscript include the following:

Supplemental Tables 1 to 5 (.xlsx)
Supplemental Movies 1 to 3 (.mp4)

Supplementary Methods:

Diagnosis of adolescent idiopathic scoliosis (AIS)

Spine surgeons performed a full clinical examination for any patient with spinal deformity. This examination includes assessment of any spinal pain, neurocutaneous stigmata such as sacral dimple and café au lait lesions, assessment of the abdominal and lower limb reflexes for any asymmetry and any upgoing Babinski's reflexes. An MRI was obtained for any abnormal signs noted above and if the curve is presented before the age of 10. For a radiographic assessment, a left-sided thoracic curve, thoracic lordosis, vertebral malformations, short angular curves, or rapidly progressive curvatures is atypical curve and warrants an MRI to identify any neuroaxis abnormalities. All subjects were assessed by the spine surgeons to ensure they are AIS and had no neurological abnormalities prior to recruitment for this study.

Genome sequencing

Genomic DNA was isolated from peripheral blood samples using standard methods and sequenced by Macrogen Inc, Korea. For AIS families, we applied whole genome sequencing (WGS). Sequencing libraries were constructed with the TruSeq Nano DNA Kit. Paired-end sequencing was conducted at 30x coverage with Illumina HiSeq X Ten. For sporadic AIS patients from Hong Kong cohort, we performed whole exome sequencing (WES) at 100x depth with Illumina HiSeq 4000. Quality control of raw fastq data was carried out by FastQC and fastp (1, 2). Clean reads were aligned to the UCSC hg38 reference genome using Burrows-Wheeler Aligner (BWA) v0.7.17 algorithm (3). Variant calling was performed using the best practices of workflows of Genome Analysis Toolkit (GATK v4.1.0.0) (4).

Linkage analysis

Nonparametric linkage analyses of the two large families (Family 1 and Family 2) were performed using the "--npl" parameter in MERLIN, under the Kong and Cox exponential model (5, 6). The output interval was 5 centimorgans along the chromosomes. A logarithm of the odds (LOD) score greater than 3 was considered evidence of linkage.

Variant filtering and annotation

After variant calling, all exonic variants that follow Mendelian inheritance (Dominant Model) were extracted. The extracted variants were annotated with external databases (SIFT, Polyphen, LRT, Mutation Taster, Mutation Assessor, FATHMM, M-CAP, GERP and CADD) using ANNOVAR (7). These variants were further filtered based on the following criteria: 1) non-synonymous coding variant, 2) obey Mendelian laws of inheritance, 3) shared by affected individuals but not present in unaffected individuals, 4) minor allele frequency less than 0.01 in gnomAD database, and 5) annotated as deleterious by more than one resource.

Targeted sequencing

The primers used for R662W and Y206F variants targeted sequencing are as follows:
R662W-Forward: 5'-GTGATCCAGTAC CAGCCGAT-3'
R662W-Reverse: 5'-ACTAGCAGTGGTGACCAAGG-3'

Y206F-Forward: 5'-CTGGATGCTGAGGCTCCTC-3'
Y206F-Reverse: 5'-GCTCTTCTCCTAA ACTCTTC-3'
PCR products were subjected to Sanger sequencing. The above primers were used in targeted sequencing for 725 sporadic AIS patients and 3,219 controls of Hong Kong population.

DNA construct
The wild type human *SLC6A9* coding sequence was synthesized and subcloned into pCMV-3tag-1A plasmid at EcoRI and XhoI sites. The HA-tagged SLC6A9 wild type plasmid was generated by replacing the 3 X Flag sequence with 3 X HA. The various GLYT1 variants were introduced by site-directed mutagenesis (Fast MultiSite Mutagenesis System, Transgen, China). The internal transfection control plasmid expressing HA-tagged Vangl2 was described previously (8). A plasmid containing *Tol2* coding sequence, pT3TS-Tol2 (a gift from Stephen Ekker, Addgene plasmid #31831), was used to produce *Tol2* Transposase mRNA in vitro (mMESSAGE mMACHINE™ T3 Transcription Kit, ThermoFisher Scientific, #AM1348, U.S.). To visualize neuronal activities in zebrafish larvae, the Tol2-elavl3-H2B-GCaMP6s plasmid (a gift from Misha Ahrens, Addgene plasmid #59530) that expresses nuclear-localized GCaMP6-slow, a calcium indicator, was microinjected into one-cell stage zygotes of zebrafish with *Tol2* mRNA.

Transfection
All cell lines used in this study were routinely tested as mycoplasma negative. HEK293T and MDCK cells were cultured in Dulbecco's Modified Eagle's Medium (DMEM, Invitrogen, #12800017, U.S.) supplemented with 10% Fetal Bovine Serum (FBS, Gibco, #10099141, U.S.), 100 units/mL of penicillin G and 100 μg/mL of streptomycin (ThermoFisher Scientific, #15140163, U.S.) at 37°C in a humidified incubator with 5% CO_2. HEK293T cells were transfected with indicated plasmids using 1 μg/μL of polyethylenimine (PEI, Sigma Aldrich, #765090, U.S.) at the ratio of plasmid:PEI = 1:3. MDCK cells were transfected with indicated plasmids using Lipofectamine 3000 Transfection Reagent following manufacturer's instruction (ThermoFisher Scientific, #L3000015, U.S.). Transfected cells were harvested for immunoblot or immunofluorescence analysis 48 hours after transfection.

Cell surface biotinylation assay
HEK293T cells were plated onto 6-well plates at 70%-80% confluence and transfected with indicated plasmids, as well as an unrelated membrane protein HA-tagged Vangl2 as an internal transfection control. 48 hours after transfection, cells were rinsed with ice-cold PBS three times and incubated for 30 minutes at 4°C in 0.5 mL PBS containing the 1 mg/mL non-permeable EZ-Link™ Sulfo-NHS-SS-Biotin reagent (ThermoFisher Scientific, #21331, U.S.). The cells were then incubated with 100 mM L-lysine (Sigma Aldrich, #L5501, U.S.) in PBS for 30 minutes at 4°C to quench the biotinylation reaction. After three additional washing with ice-cold PBS, the cells were lysed with 1 mL of modified RIPA lysis buffer (150 mM NaCl, 5 mM EDTA, 25mM Tris, 0.5% deoxycholate, 1%Triton X-100, 1% NP-40 and 0.1% SDS, pH 7.4) for 30 minutes on ice. Cell lysates were cleared by centrifugation at 14000 rpm at 4°C for 30 minutes, and 200 μL of cell lysates were collected and served as total cell lysate fraction. The biotinylated proteins were recovered by incubating the

remaining cell lysates for 2 hours at room temperature with Pierce™ high-capacity streptavidin agarose resin (ThermoFisher Scientific, #20359, U.S.). After three washing of the agarose resin with 1 mL of lysis buffer, the biotinylated proteins were eluted with 2X Laemmli sample buffer (4% SDS, 20% Glycerol, 120 mM Tris, 0.02% bromophenol blue, 100mM dithiothreitol, pH 7.4). Total cell lysates and the biotinylated cytomembrane fraction were stored at -80°C until further use.

Antibodies and secondary antibodies

Antibodies	Vendor	Catalog. No.	WB	IF
HA	Roche	11867431001	1:5000	1:1000
	CST	C29F4, 3724		1:1000
FLAG	Sigma-Aldrich	M2, F3165	1:5000	1:1000
Actin	Sigma-Aldrich	A2228	1:10000	
F59	DSHB	MYH1A		1:100
Anti-acetylated α Tubulin	Millipore Sigma	T7451		1:300
HRP-conjugated mouse	GE Healthcare	NA9310	1:10000	
HRP-conjugated rabbit	GE Healthcare	NA9340	1:10000	
Alexa Fluor 488 donkey anti rabbit IgG	ThermoFisher Scientific	A32790		1:1000
Alexa Fluor 488 donkey anti mouse IgG	ThermoFisher Scientific	A32766		1:1000
Alexa Fluor 568 donkey anti rabbit IgG	ThermoFisher Scientific	A10042		1:1000
Alexa Fluor 568 donkey anti mouse IgG	ThermoFisher Scientific	A10037		1:1000

Western blotting

The harvested protein samples were loaded on SDS-PAGE gel (4% stacking and 8% or 10% separating gels). The samples were transferred to polyvinylidene difluoride membranes (PVDF, Merck, #IPVH00010, U.S.) by a wet western blot transfer apparatus and then blocked with 5% Bovine Serum Albumin (BSA, Sigma Aldrich, #A3912, U.S.) in PBS containing 0.1% Tween 20. The PVDF membrane was incubated with indicated primary antibodies overnight at 4°C and then secondary antibodies for 2 hours at room temperature. Super Signal™ West Pico PLUS Chemiluminescent Substrate (ThermoFisher Scientific, #34580, U.S.) was used to detect immunoblot signals.

Immunofluorescence and confocal microscopy

MDCK cells were seeded onto slides in 24 well plates and grown to 50%-60% confluence. Cells were transfected with indicated plasmids and incubated for 48 hours before fixation. Cells were rinsed three times with cold PBS and then fixed with 4% paraformaldehyde (PFA, Sigma Aldrich, #158127, U.S.) in PBS at room temperature for 10 minutes, followed by permeabilization with 0.5% Triton X-100/PBS for 5 minutes. After washing with 0.1% Triton X-100/PBS twice, cells were blocked with 3% BSA in 0.1% Triton X-100/PBS for 1 hour at room temperature, and incubated with indicated primary antibodies overnight at 4°C. After rinsing 3 times for 5 minutes each with 1 X PBS with 0.1% Triton X-100, secondary antibodies coupled to Alexa Fluor 488 or 568 (1:1000, Life Technologies, U.S.) were applied at the room temperature for 1 hour. After rinsing 3 times with 1 X PBS with 0.1% Triton X-100, slides were mounted with ProLong Diamond Antifade Mountant with DAPI (ThermoFisher Scientific, #P36962, U.S.) and subjected to Zeiss LSM800 confocal microscopy. Images were analyzed by Zeiss Zen software. ImageJ software was used to quantify the cytomembrane and cytosolic signals of GLYT1. Signal intensities of the entire cell and intracellular fluorescence were respectively measured after calibration of background signals around the cell. The membrane signal intensities were calculated by subtracting the intracellular intensities from the total cell intensities.

Zebrafish brain disassociation for extracellular glycine level measurement

At 7 dpf, zebrafish embryos were anesthetized in 0.01% MS222 (pH=7.0, Sigma Aldrich, #10521, U.S.) in 1X PBS. The fish's whole brain was dissected out under a stereo microscope by using pairs of ultra fine forceps. Ten brains were collected in one tube and PBS was removed by low-speed centrifugation (1500 rpm for 5 minutes at 4 °C). 100 μL TrypLE™ Express Enzyme (1X, ThermoFisher Scientific, #AC 12604013, U.S.) was added to dissociate brains, followed by a 10-minute incubation in a 37 °C water bath. Gentle pipetting up and down was applied to facilitate dissociation, and the reaction was stopped by adding 100 μL of ddH_2O. The sample was then centrifuged at 1500 rpm for 5 minutes at 4 °C, and the cell-free supernatant was collected as the extracellular fluid of the zebrafish. The glycine concentration was measured using the fluorometric glycine assay kit (Abcam, #ab211100, UK), according to the manufacturer's instructions. Fluorescence was measured at Ex/Em 535/587nm in endpoint mode using a microplate reader (Varioskan Flash, ThermoFisher Scientific, U.S.).

Antibody staining and imaging of zebrafish

The fertilized eggs were collected in the morning and cultured in E3 medium at 28.5 °C for 12 hours, and then 1-phenyl 2-thiourea (PTU, ThermoFisher Scientific, #AC207250050, U.S.) was added into E3 medium at a final concentration 0.003% to prevent pigmentation. At 24 hours post-fertilization (hpf), the unhatched embryos were manually dechorionated using ultra-fine-tip forceps under a stereomicroscope. The embryos were fixed with 4% PFA in PBS for 1-2 hours at room temperature, followed by washing three times in 1% Triton-X/PBS for 5 minutes each. The fixed embryos were blocked in 10% goat serum (Sigma Aldrich, #G9023, U.S.) in 1% Triton-X/PBS for 1-2 hours at room temperature and were then incubated with primary antibody diluted in blocking solution overnight at 4 °C with rotation.

Next day, the embryos were washed five times in 1% Triton-X/PBS for 10 minutes each at room temperature with rocking, and then incubated with secondary antibody diluted in blocking solution for 2 hours at room temperature (or overnight at 4 °C). After three times washing of 1% Triton-X/PBS, the embryos were embedded with 0.8-1% low-melting agarose gel (SeaPlaque™ Agarose, Lonza Bioscience, #50101, U.S.) and then transferred onto the 6 cm confocal dishes with desired direction, which were later subjected to Zeiss LSM800 confocal microscopy. Images were analyzed by Zeiss Zen software.

To quantify the total level of activated spinal cord neurons in wild-type and *slc6a9*$^{m/m}$ mutant zebrafish, embryos with a *Tg(elavl3-H2B-GCaMP6s)* transgenic background were collected at 24 hpf and fixed in 4% PFA in 1X PBS for 1-2 hours at room temperature. After three washes with 1X PBS, the embryos were embedded in 0.8-1% low-melting agarose gel and transferred onto 6 cm confocal dishes with the desired orientation. Snap captures of the dorsal view were obtained for both wild-type and *slc6a9*$^{m/m}$ mutant embryos using the same confocal settings (Laser wavelength: 488nm; 0.94%; Pinhole: 32µm; Detector Gain: 700V) in a Zeiss LSM800 confocal microscope. The images were analyzed using ImageJ software, with the spinal cord neuronal GCaMP6s signals used for quantification.

Live staining and imaging of zebrafish

Fertilized zebrafish eggs were collected in the morning and cultured in E3 medium until the desired developmental stage. Live staining was performed on zebrafish embryos using vital dyes to visualize cell internal membranes and acidic notochord vacuoles. Specifically, 48 hpf wild-type and mutant embryos were stained with 100 µM BODIPY TR Methyl Ester (MED, Invitrogen, # C34556, U.S.) for 1 hour, followed by three washes with E3 medium. At 6 dpf, LysoTracker GreenDND-26 (Invitrogen, # L7526, U.S.) was used to live-label acidic notochord vacuoles. Wild-type and mutant zebrafish were stained with 50 µM LysoTracker GreenDND-26 for 2 hours, followed by three washes with E3 medium. The stained embryos were anesthetized with MS222, mounted in 0.8-1% low-melting agarose gel, and transferred onto 6 cm confocal dishes with the desired orientation. Confocal imaging was performed using a Zeiss LSM800 confocal microscope, and images were analyzed with Zeiss Zen software. Zebrafish live skeletal staining was performed by incubating embryos in a 0.2% Calcein (pH 7.0, Sigma Aldrich, #C0875, U.S.) solution for 10 minutes, followed by three washes with E3 medium. Calcein-stained embryos were imaged using an Olympus IX71 microscope (Olympus, Japan).

Zebrafish mRNA microinjection

The zygotes from *slc6a9*$^{m/+}$ and *slc6a9*$^{m/+}$ mating pairs were used for mRNA microinjection. The *SLC6A9* mRNA encoding human wild type, Y206F or R662W variant GLYT1 was synthesized using the mMESSAGE mMACHINE™ T3 Transcription Kit (ThermoFisher Scientific, #AM1348, U.S.). 60, 120, 200, and 400 pg of wild type *GLYT1* mRNA were respectively injected into the zygotes to test the optimal rescue dosage. As the injection of 200 pg of wild type *GLYT1* mRNA significantly rescued the curvature phenotype of *slc6a9*$^{m/m}$ fish, the same dosage of Y206F and R662W variant mRNA was used for rescue experiments. At 7 dpf, all fish were examined for axial phenotype and then lysed for genotyping.

Swimming behavior

At 7 dpf, zebrafish larvae were individually transferred into 48-well plate. The swimming distance and trajectory over 10 minutes was recorded under Leica MZ10F Fluorescence Stereomicroscope with a vision recording system. Analysis was performed by using idTracker in MATLAB (9). After recording, all the fish were sacrificed for genotyping.

Reference:

1. Wingett SW, and Andrews S. FastQ Screen: A tool for multi-genome mapping and quality control. *F1000Res.* 2018;7:1338.
2. Chen S, Zhou Y, Chen Y, and Gu J. fastp: an ultra-fast all-in-one FASTQ preprocessor. *Bioinformatics.* 2018;34(17):i884-i90.
3. Li H, and Durbin R. Fast and accurate short read alignment with Burrows-Wheeler transform. *Bioinformatics.* 2009;25(14):1754-60.
4. Van der Auwera GA, Carneiro MO, Hartl C, Poplin R, Del Angel G, Levy-Moonshine A, et al. From FastQ data to high confidence variant calls: the Genome Analysis Toolkit best practices pipeline. *Curr Protoc Bioinformatics.* 2013;43(1110):11 0 1- 0 33.
5. Ott J, Wang J, and Leal SM. Genetic linkage analysis in the age of whole-genome sequencing. *Nat Rev Genet.* 2015;16(5):275-84.
6. Abecasis GR, Cherny SS, Cookson WO, and Cardon LR. Merlin--rapid analysis of dense genetic maps using sparse gene flow trees. *Nat Genet.* 2002;30(1):97-101.
7. Wang K, Li M, and Hakonarson H. ANNOVAR: functional annotation of genetic variants from high-throughput sequencing data. *Nucleic Acids Res.* 2010;38(16):e164.
8. Feng D, Wang J, Yang W, Li J, Lin X, Zha F, et al. Regulation of Wnt/PCP signaling through p97/VCP-KBTBD7–mediated Vangl ubiquitination and endoplasmic reticulum–associated degradation. *Science advances.* 2021;7(20):eabg2099.
9. Pérez-Escudero A, Vicente-Page J, Hinz RC, Arganda S, and De Polavieja GG. idTracker: tracking individuals in a group by automatic identification of unmarked animals. *Nature methods.* 2014;11(7):743-8.

Captions for Supplemental Tables 1 to 5.

(Supplemental Tables 1 to 5 are available in Excel document format in Other Supplementary Materials)

Supplemental Table 1. Phenotype of participants in this study.

Supplemental Table 2. Genetic variants segregating with AIS phenotype in Family 1 and 2.

Supplemental Table 3. Summary of *SLC6A9* variants identified in AIS patients.

Supplemental Table 4. Summary of p.Y206F variant carriers in the studied population.

Supplemental Table 5. The p.Y206F variant in different populations.

Captions for Supplemental Movies 1 to 3.

(Supplemental Movies 1 to 3 are available in MP4 format in Other Supplementary Materials)

Supplemental Movie 1. Wild type zebrafish at 7dpf
Supplemental Movie 2. The phenotype of *slc6a9* mutant zebrafish #1 at 7dpf
Supplemental Movie 3. The phenotype of *slc6a9* mutant zebrafish #2 at 7dpf.

Supplemental Figure 1

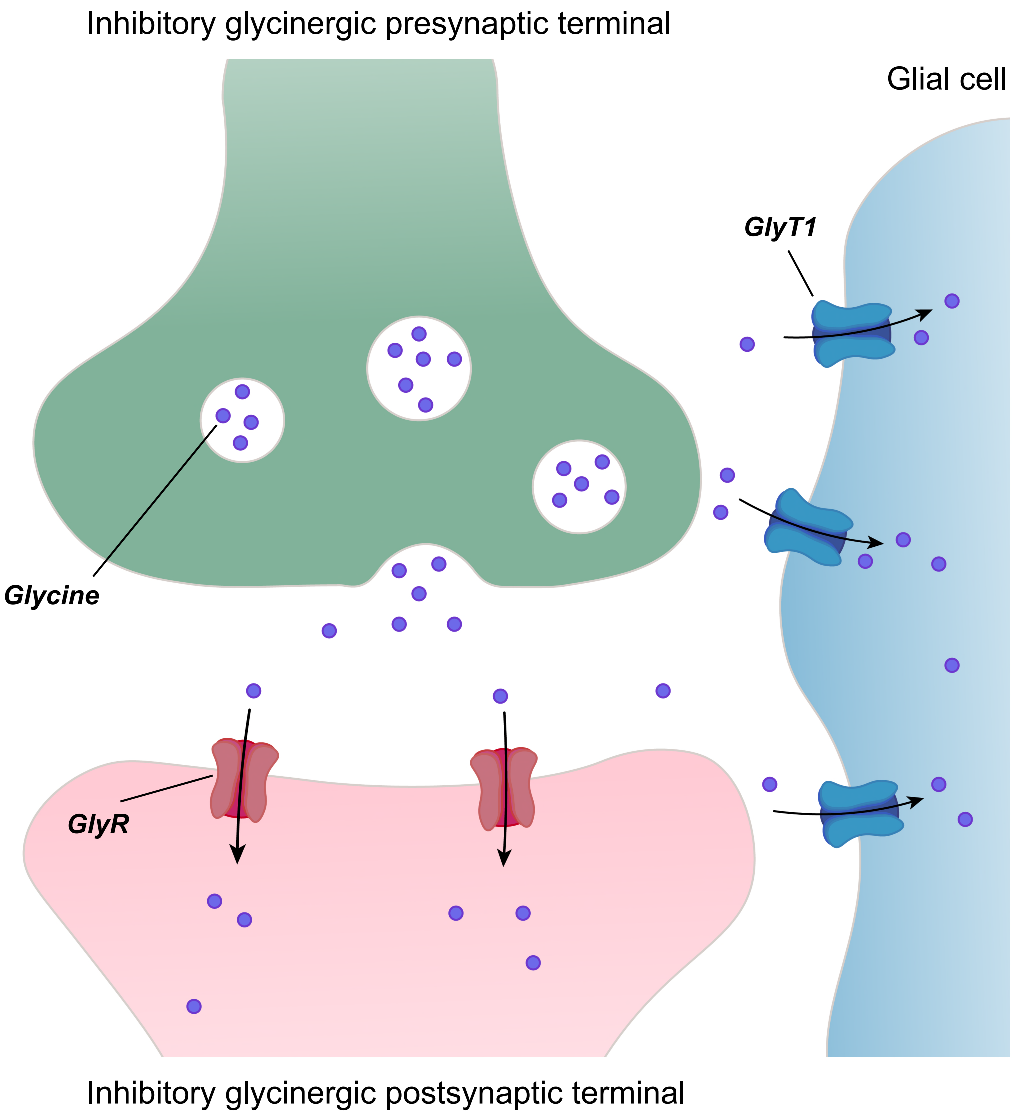

Supplemental Figure 1. Schematic representation of GlyT1 function in inhibitory glycinergic synapse. Glycine is released from inhibitory glycinergic presynaptic terminal and binds to Glycine receptors (GlyRs) on postsynaptic membrane. GlyT1 is primarily expressed on astrocytes surrounding glycinergic synapses to facilitate rapid clearance of glycine from the synaptic cleft.

Supplemental Figure 2

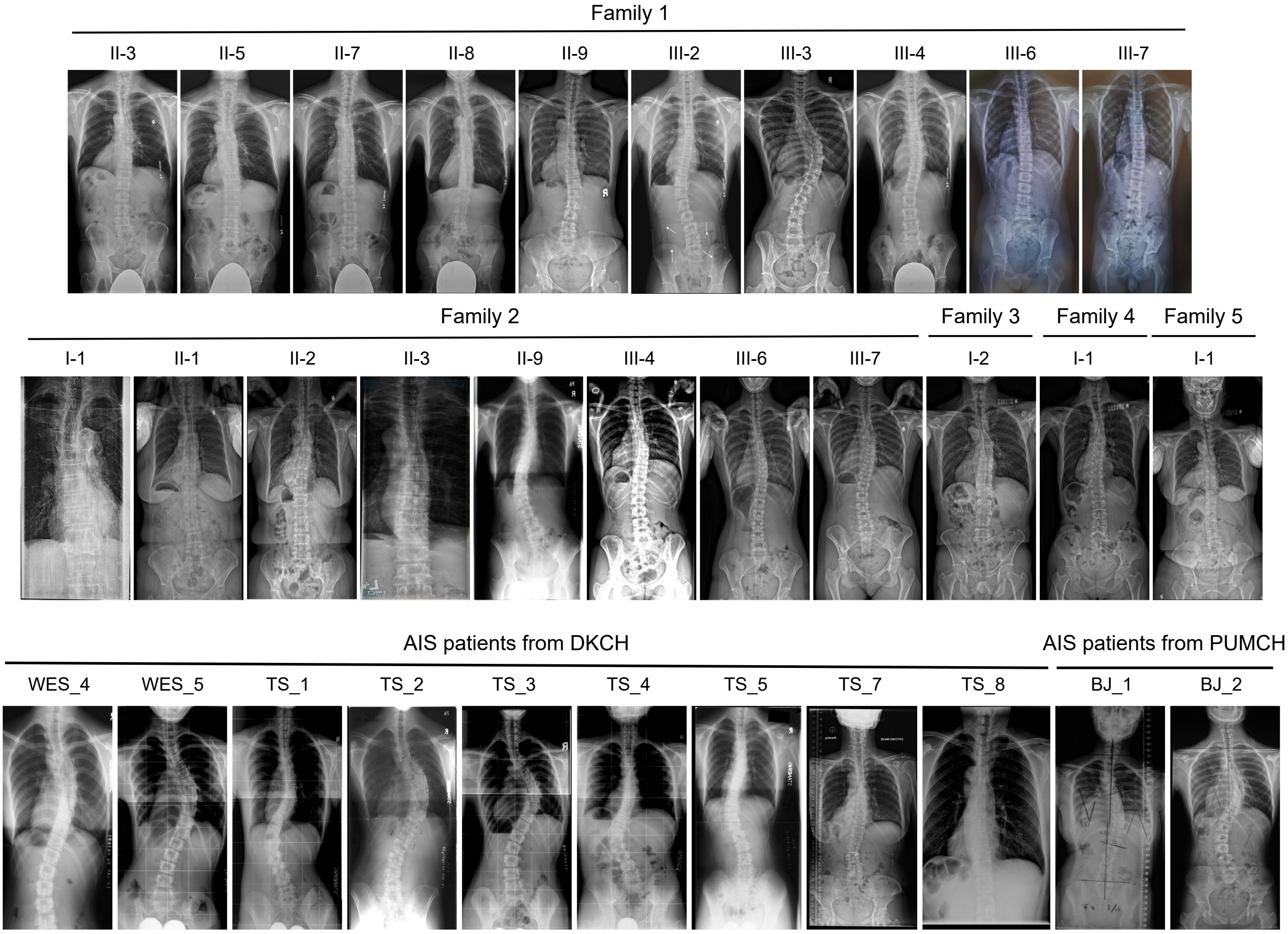

Supplemental Figure 2. Spinal radiographs of familial and sporadic AIS patients. Patients were subjected to full spine X-ray scanning in a relaxed and standing posture. The posteroanterior whole spine radiographs are shown. DKCH: Duchess of Kent Children's Hospital; PUMCH: Peking Union Medical College Hospital.

Supplemental Figure 3

A

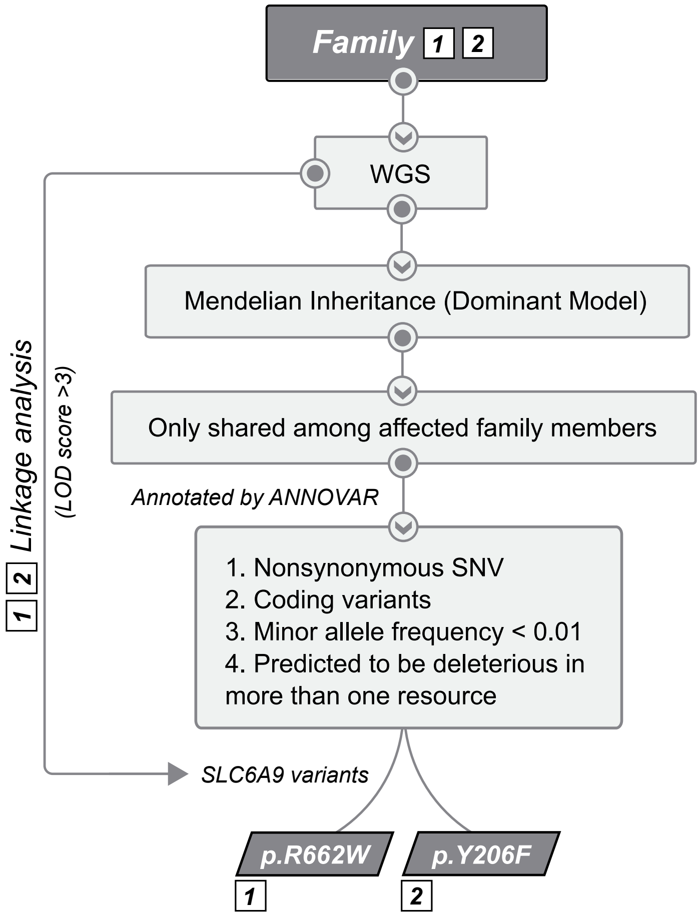

B

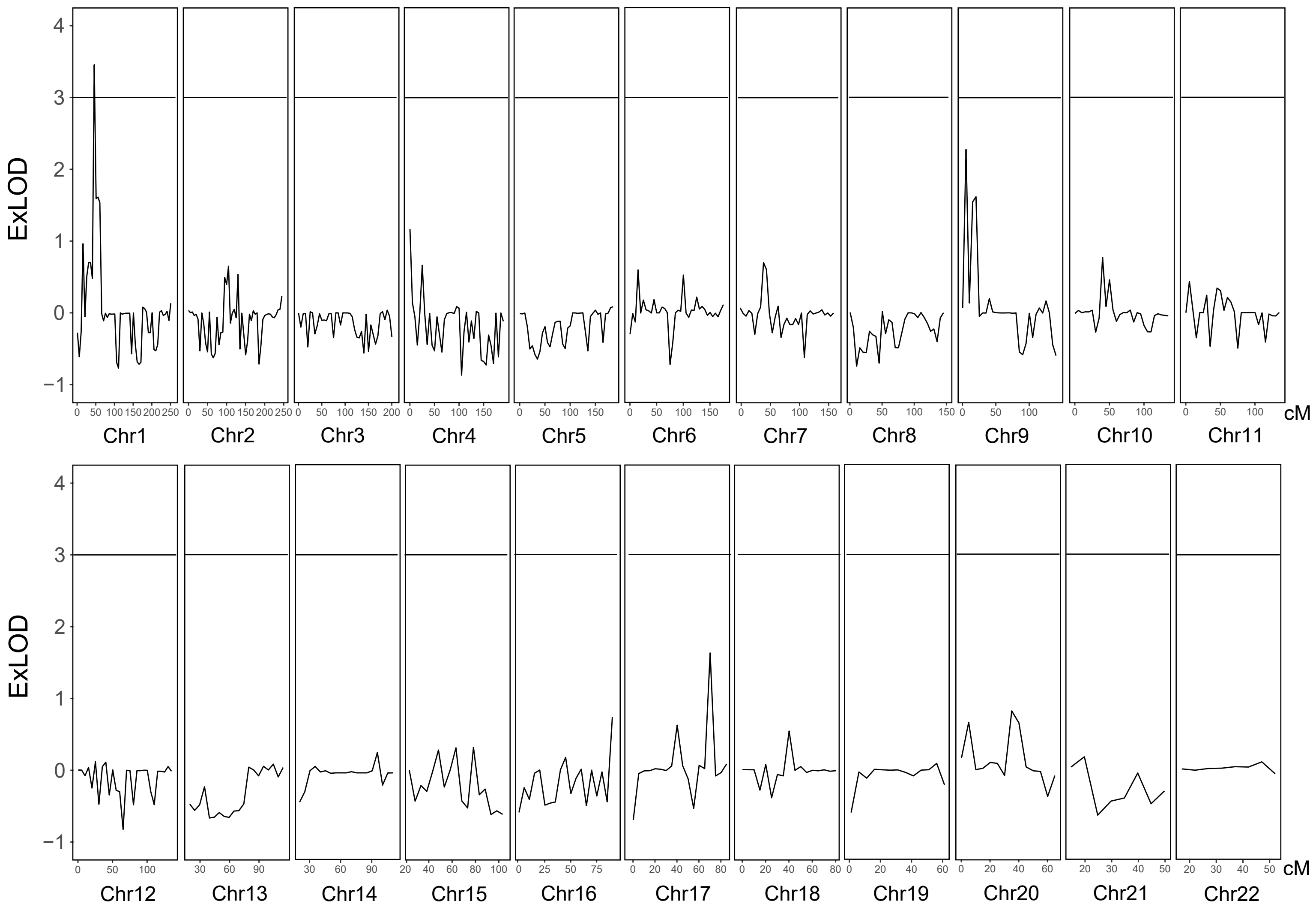

Supplemental Figure 3. Genetic analysis of AIS Family 1 and 2. (**A**) Workflow of genetic analysis. WGS data of Family 1 and 2 were analyzed independently. Linkage analysis identified a locus in chromosome 1 containing *SLC6A9*, in which two *SLC6A9* variants, c.1984C>T, p.R662W and c.617A>T, p.Y206F are associated with Family 1 and 2, respectively. (**B**) Linkage analysis of Family 1 and 2. The X-axis represents the relative location on the chromosome. One single centimorgan (cM) is approximately equal to 1 million base pairs (bps). The Y-axis represents the exponential LOD (logarithm of the odds) scores from nonparametric linkage analyses.

Supplemental Figure 4

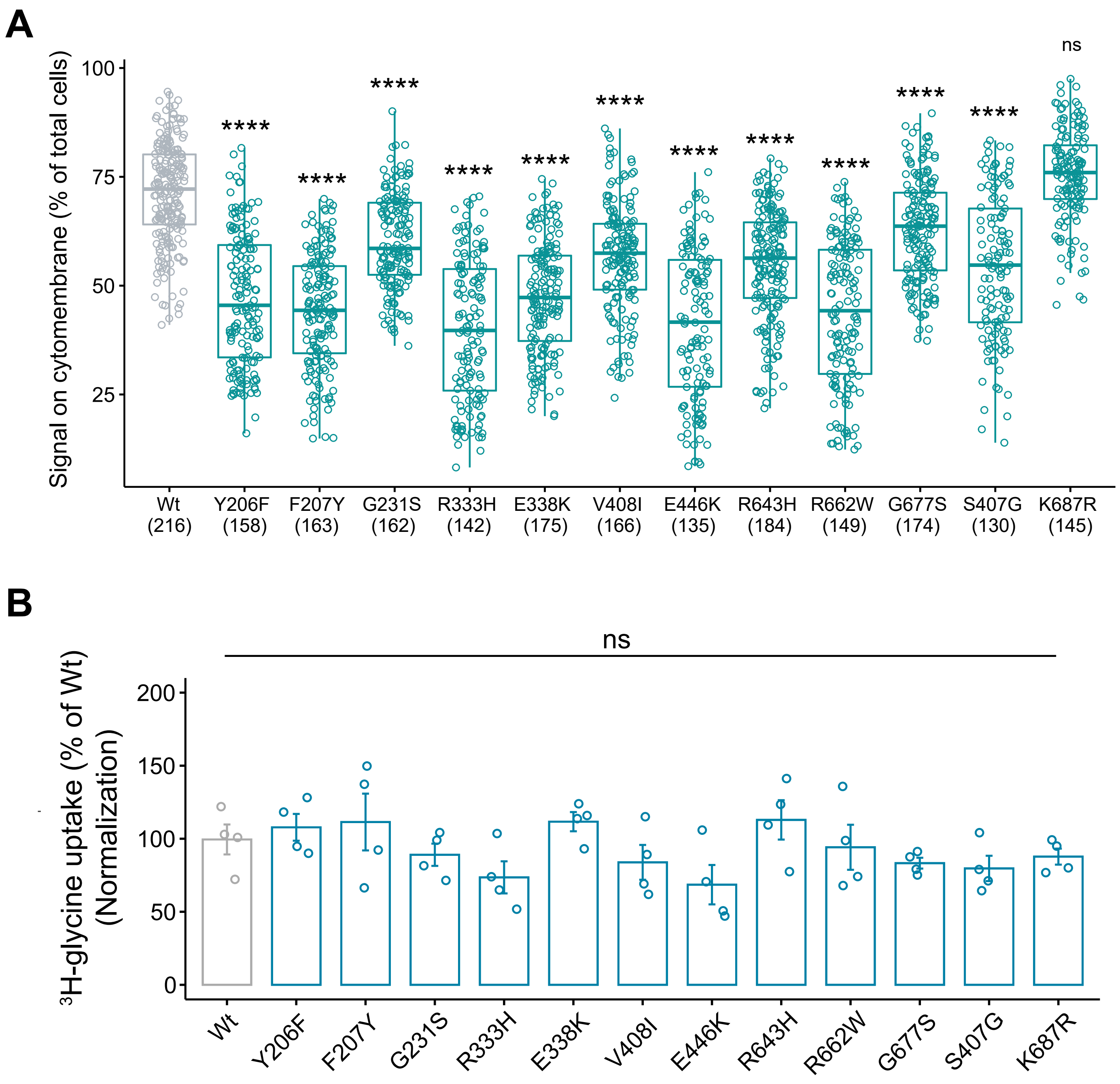

Supplemental Figure 4. Quantification of functional assays of GLYT1 variants. (**A**) Statistical ratio of cytomembrane to total cell signal intensities of GLYT1 wild type and variants. The number of calculated cells of each group is indicated on the bottom of each box. Boxes show the median and IQRs with all individual data points superimposed. 1-way ANOVA test, ****P < 0.0001; ns, no significance. (**B**) The normalized glycine uptake activities of GLYT1 variants. Glycine uptake activities of *SLC6A9* variants were normalized to cytomembrane protein expression levels. Each data dot represents one independent experiment. Data are shown as means ±SEM. 1-way ANOVA test. ns, no significance.

Supplemental Figure 5

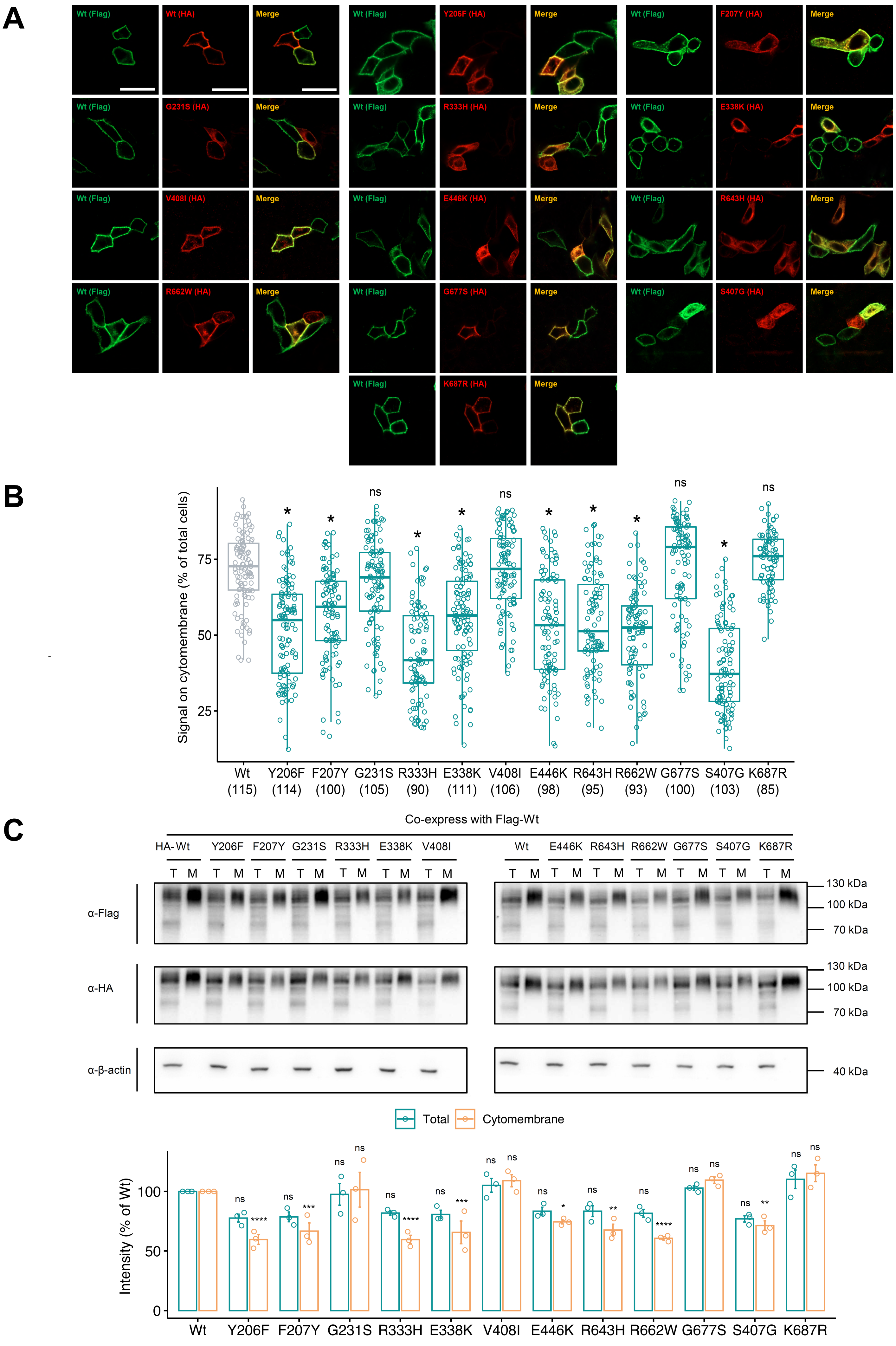

Supplemental Figure 5. Dominant negative effects of GLYT1 variants over wild type. (**A**) Subcellular localization of GLYT1 wild type when co-expression with various GLYT1 variants. Confocal microscopy of transfected MDCK cells showing co-expression of Flag-GLYT1 wild type and HA-GLYT1 wild type or variants. Signals were visualized with anti-Flag antibody (green) and anti-HA antibody (red). Scale bar = 20μm. (**B**) Quantification of cytomembrane localization of Flag-GLYT1 wild type in (A). Statistical results indicate the percentage of cytomembrane signals of Flag-GLYT1 wild type in co-expression cells. The number of calculated cells of each group was shown on the bottom of each box. Boxes show the median and IQRs with all individual data points superimposed. 1-way ANOVA, *$p<0.05$; ns, no significance. (**C**) Immunoblots of GLYT1 wild type when co-expression with GLYT1 wild type or variants. Western blot analysis of HEK293T total cell extracts (T) and cytomembrane fractions (M) after transient expression of Flag-GLYT1 wild type with HA-GLYT1 wild type or variants. β-actin was used as a loading control for total cell lysates. Quantification of immunoblots in (C). Each data dot represents one independent experiment. Data are shown as means ±SEM. 2-way ANOVA test, *$P < 0.05$; **$P < 0.01$; ***$P < 0.001$; ****$P < 0.0001$; ns, no significance.

Supplemental Figure 6

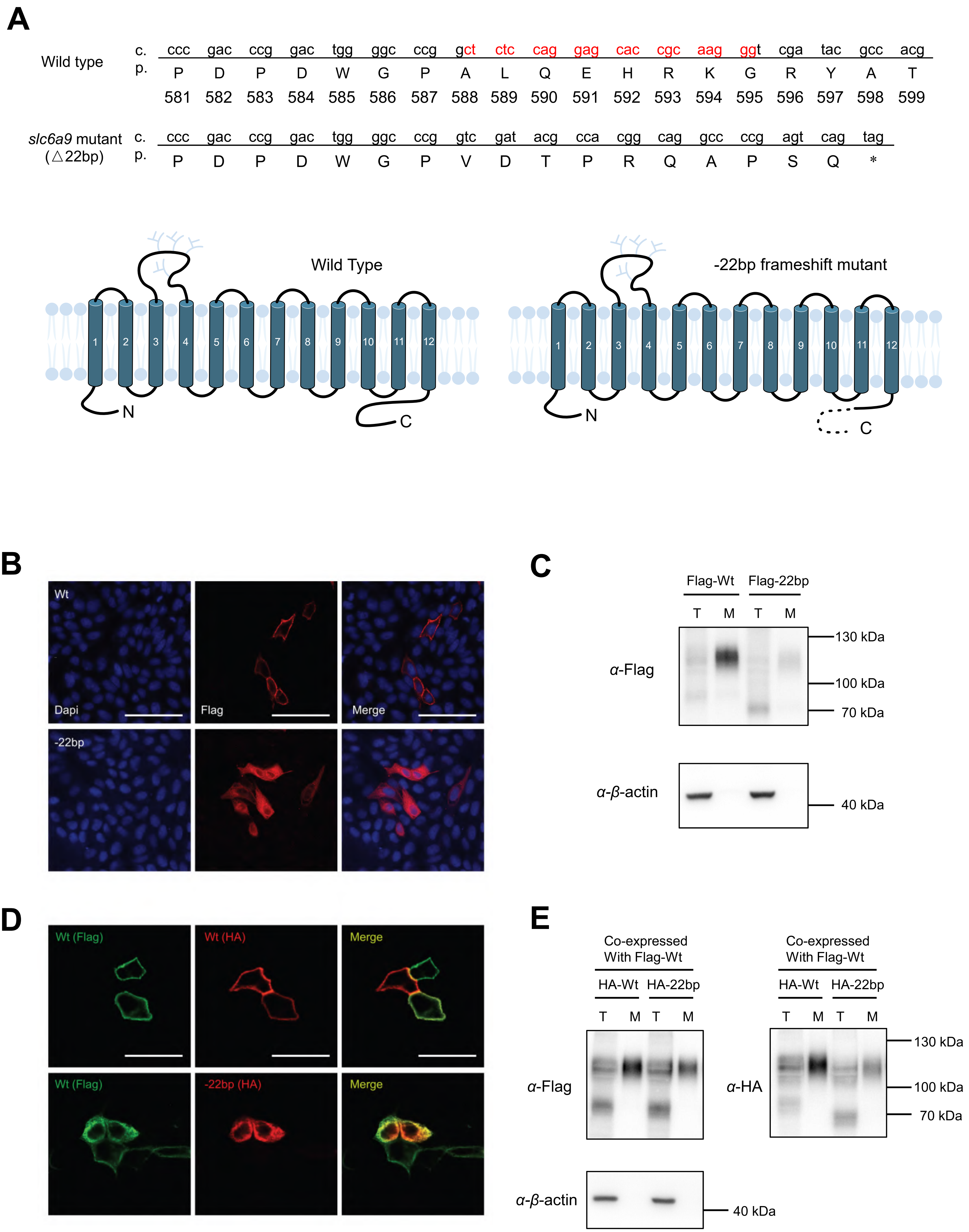

Supplemental Figure 6. Generation of *slc6a9* mutant zebrafish model. (**A**) The 22 base pairs (bp) deletion of *slc6a9* results in a frameshift and C-terminal truncated glyt1 in zebrafish. The 22bp deletion is highlighted in red. The lower panel shows the schematic structure of zebrafish glyt1 wild type and mutant. (**B**) Confocal microscopy of transfected MDCK cells showing subcellular localization of Flag-tagged GLYT1 wild type and human version -22bp mutant. Signals were visualized with anti-Flag antibody (red) and nuclei were stained with DAPI (blue). Scale bar = 20μm. (**C**) Immunoblot of HEK293T cell total extracts and cytomembrane fractions expressing GLYT1 wild type or -22bp mutant. (**D**) Subcellular localization of Flag-tagged GLYT1 wild type when co-expression with HA-tagged GLYT1 wild type or -22bp mutant. Signals were visualized with anti-Flag antibody (green) and anti-HA antibody (red). The wild type panel presented is identical to the one displayed in Supplemental Figure 5A. Scale bar = 40μm. (**E**) Immunoblots of GLYT1 wild type when co-expression of GLYT1 -22bp mutant in HEK293T cells. T, total cell lysates; M, membrane fraction. β-actin was used as a loading control for total cell lysates.

Supplemental Figure 7

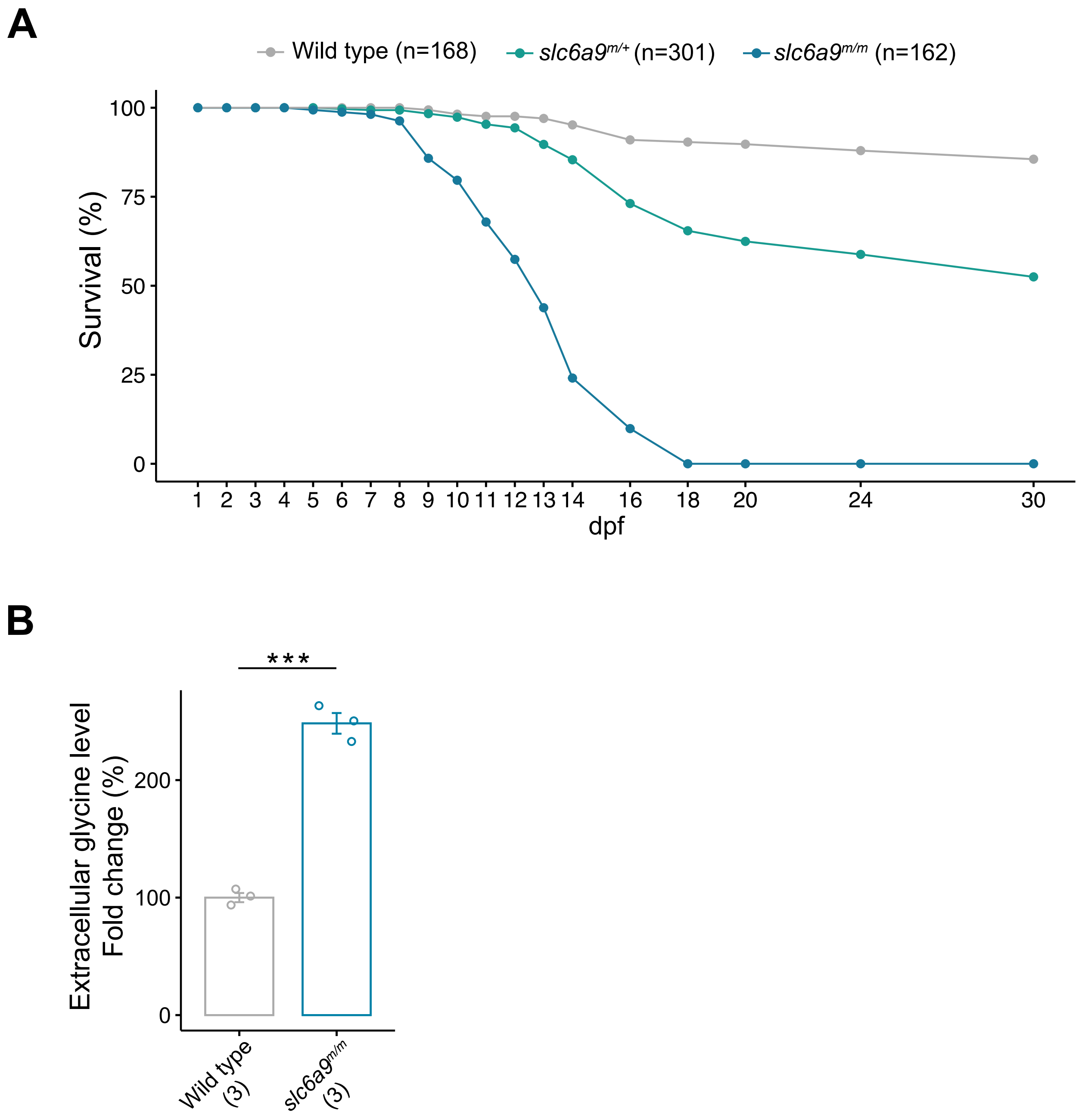

Supplemental Figure 7. Survival and glycine level of *slc6a9* mutant zebrafish. (A) The survival curve of wild type and *slc6a9* mutant zebrafish within 30 days after fertilization. Notably, all *slc6a9*$^{m/m}$ larvae die between 7 dpf and 18 dpf, whereas around 50% of *slc6a9*$^{m/+}$ and 80% wild type larvae survived to 30 dpf. **(B)** Quantification of the extracellular glycine levels in wild type and *slc6a9*$^{m/m}$ mutant zebrafish. Each data dot represents one independent experiment. Data are shown as means ±SEM. Unpaired Student *t*-test, ****P* < 0.001.

Supplemental Figure 8

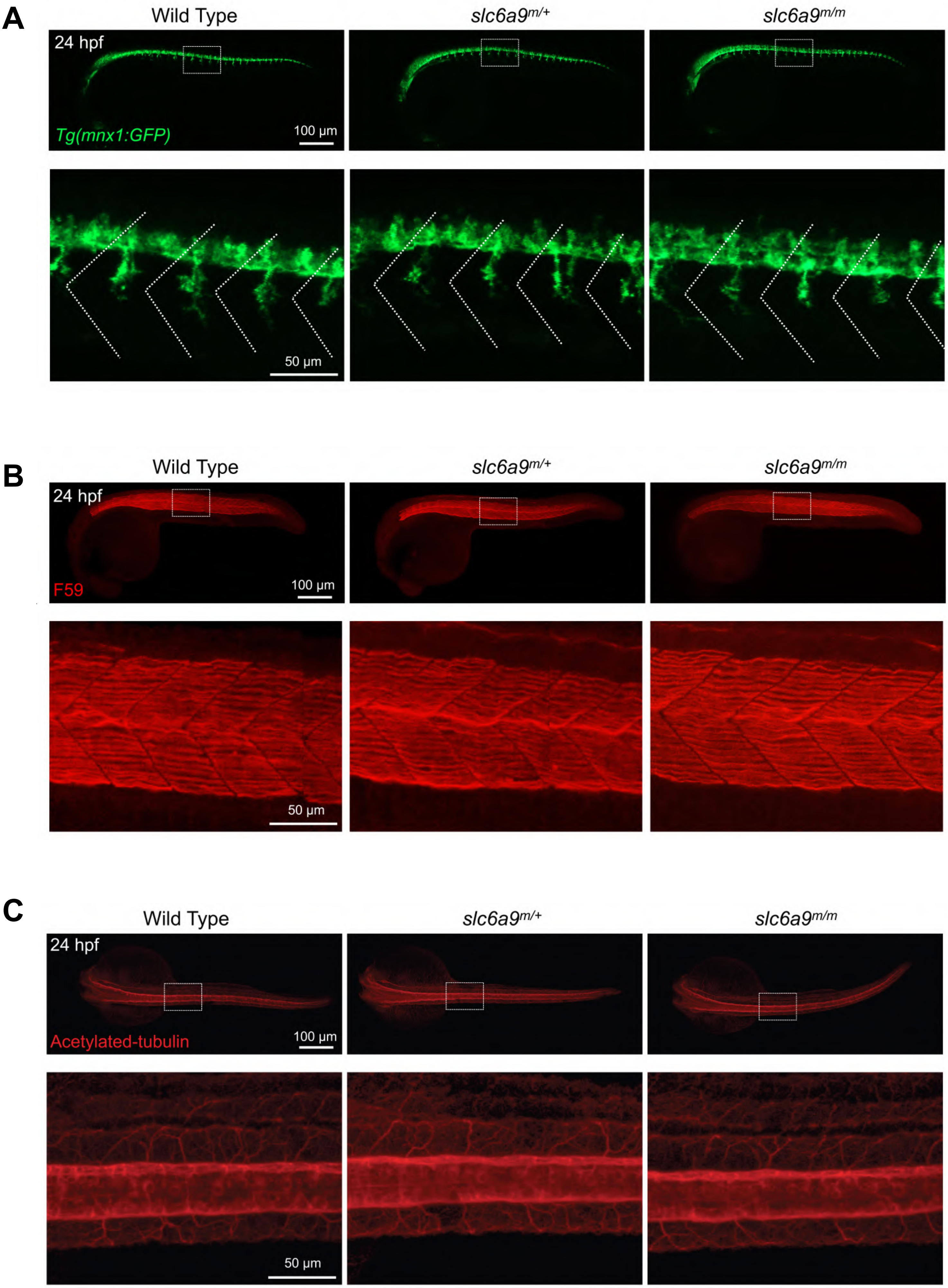

Supplemental Figure 8. *Slc6a9* mutant larvae are morphologically intact in motoneuron, skeletal muscle, and axonal tracts. (**A**) Motoneuron labeled by *Tg(mnx1:GFP)* in wild type and *slc6a9* mutant zebrafish at 24 hpf. Representative side view images of GFP signals in wild type, *slc6a9*$^{m/+}$ and *slc6a9*$^{m/m}$ larvae at 24 hpf are shown in the upper panel, and segments 9-13 are enlarged and highlighted in the lower panel. (**B**) Skeletal muscle fibers in wild type and *slc6a9* mutant zebrafish. Representative side view images of whole mount staining of F59 antibody in wild type, *slc6a9*$^{m/+}$ and *slc6a9*$^{m/m}$ larvae at 24 hpf are shown in the upper panel, and segments 11-15 are enlarged and highlighted in the lower panel. (**C**) Axonal tracts in wild type and *slc6a9* mutant zebrafish. Representative dorsal view images of whole mount staining of acetylated tubulin antibody in wild type, *slc6a9*$^{m/+}$ and *slc6a9*$^{m/m}$ larvae at 24 hpf are shown in the upper panel, and segments 10-14 are enlarged and highlighted in the lower panel.

Supplemental Figure 9

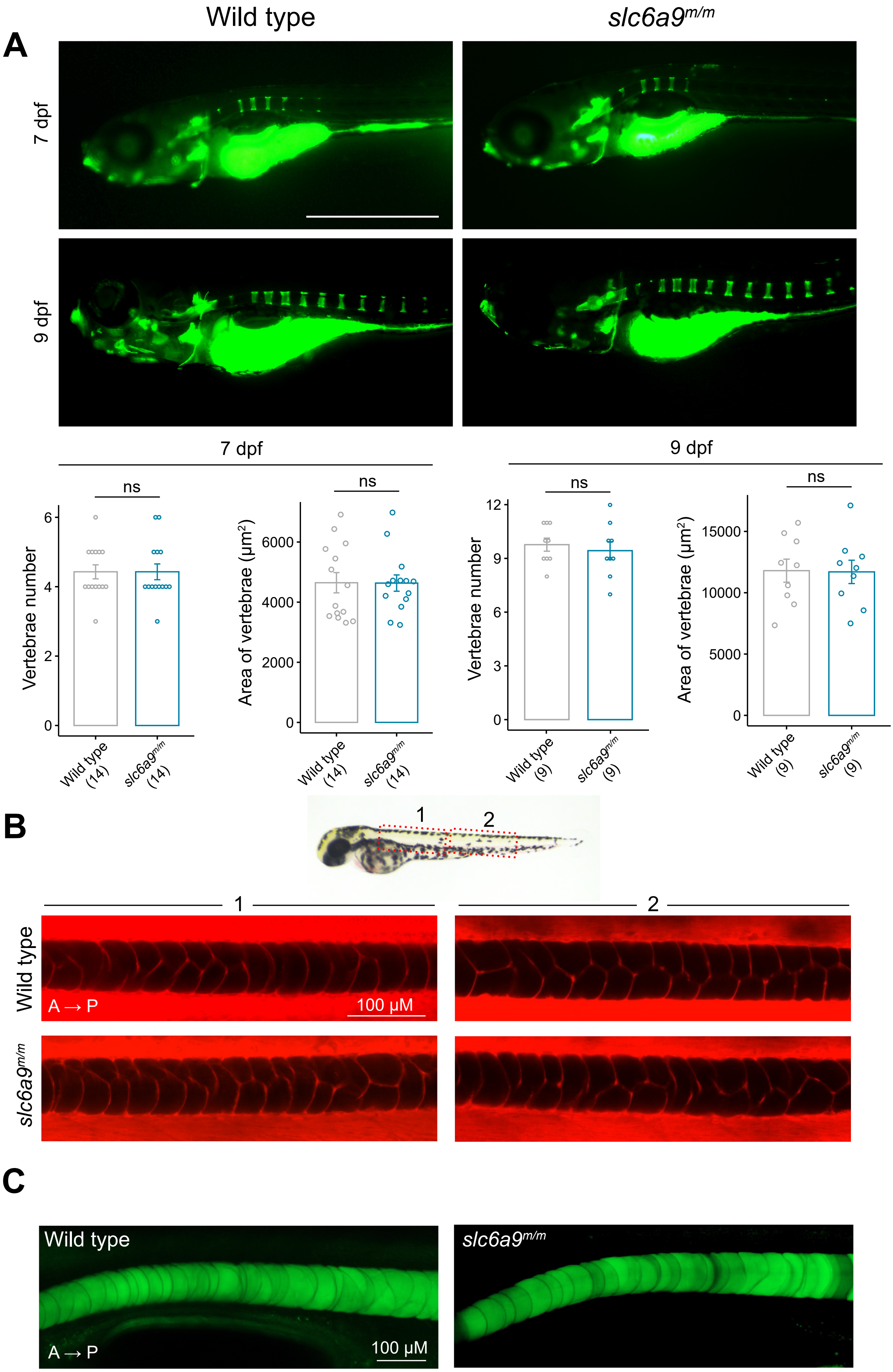

Supplemental Figure 9. ***Slc6a9*** **mutant larvae have no anomalies in vertebrae, notochord sheath, and vacuolated cells. (A)** Live imaging of 7 and 9 dpf wild type and *slc6a9*$^{m/m}$ zebrafish stained with calcein (green). Vertebrae number and area of calcified vertebrae are quantified. The number of analyzed fish is indicated for each genotype. Each dot represents one independent experiment. Error bars are 95% confidence intervals. Data are shown as means ±SEM. Unpaired Student *t*-test, ns, no significance. Scale bar indicates 0.5 mm. **(B)** Live confocal images of notochord for 48 hpf wild type and *slc6a9* mutant stained with MED to visualize internal membrane. Two regions (1 and 2) of spinal cord were captured. **(C)** Live confocal images of notochord for wild type and *slc6a9* mutant fish stained with LysoTracker Green at 6 dpf. In B and C, scale bars indicate 100 μm.

Supplemental Figure 10

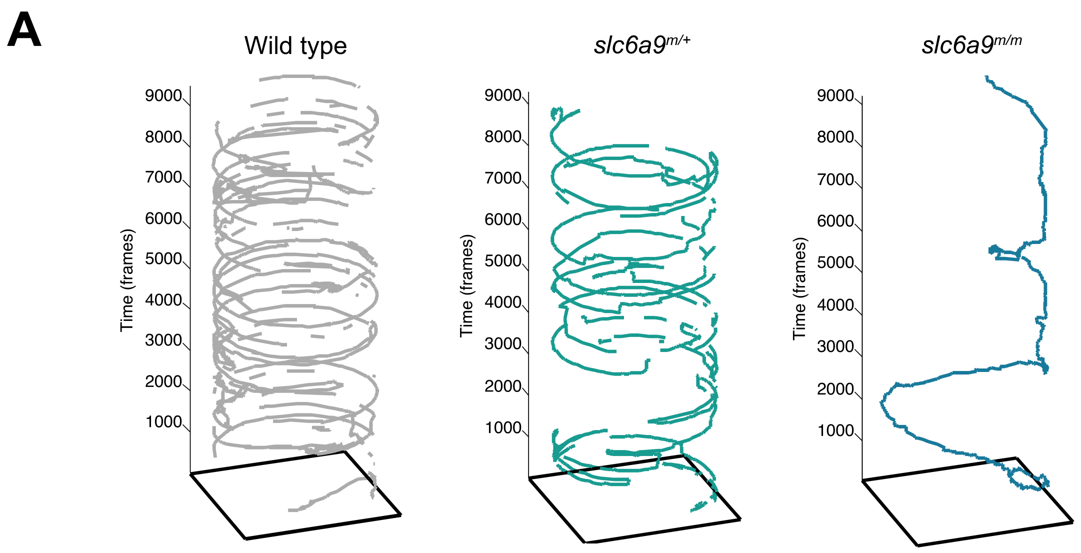

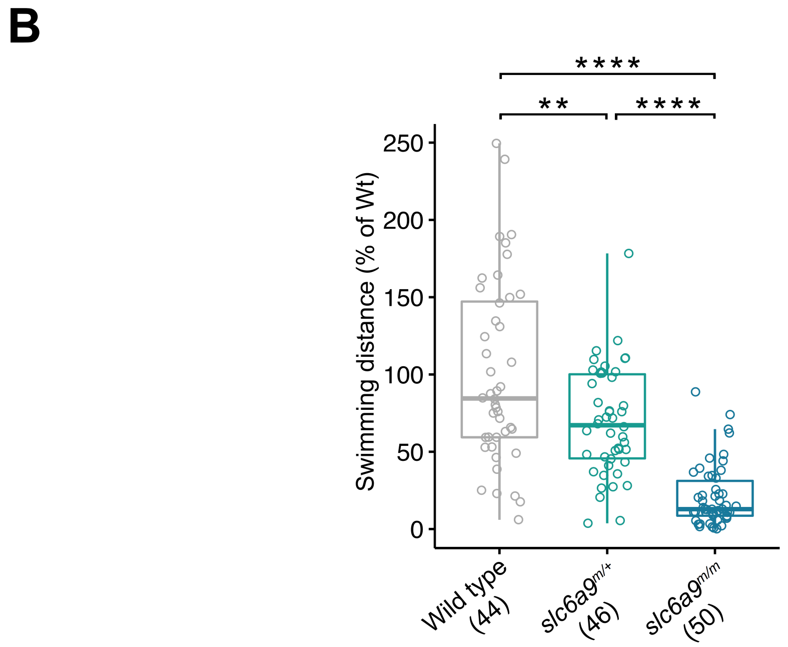

Supplemental Figure 10. Swimming behaviors in *slc6a9* mutant zebrafish. (**A**) Representative swimming tracks of wild type, *slc6a9*$^{m/+}$ and *slc6a9*$^{m/m}$ larvae at 7 dpf. (**B**) Quantification of swimming distances of wild type (n = 44), *slc6a9*$^{m/+}$ (n = 46) and *slc6a9*$^{m/m}$ (n = 50) larvae. Each data point represents the swimming distance of an individual larva. Boxes show the median and IQRs with all individual data points superimposed. 1-way ANOVA test, **$P < 0.01$; **** $P < 0.0001$.

Supplemental Figure 11

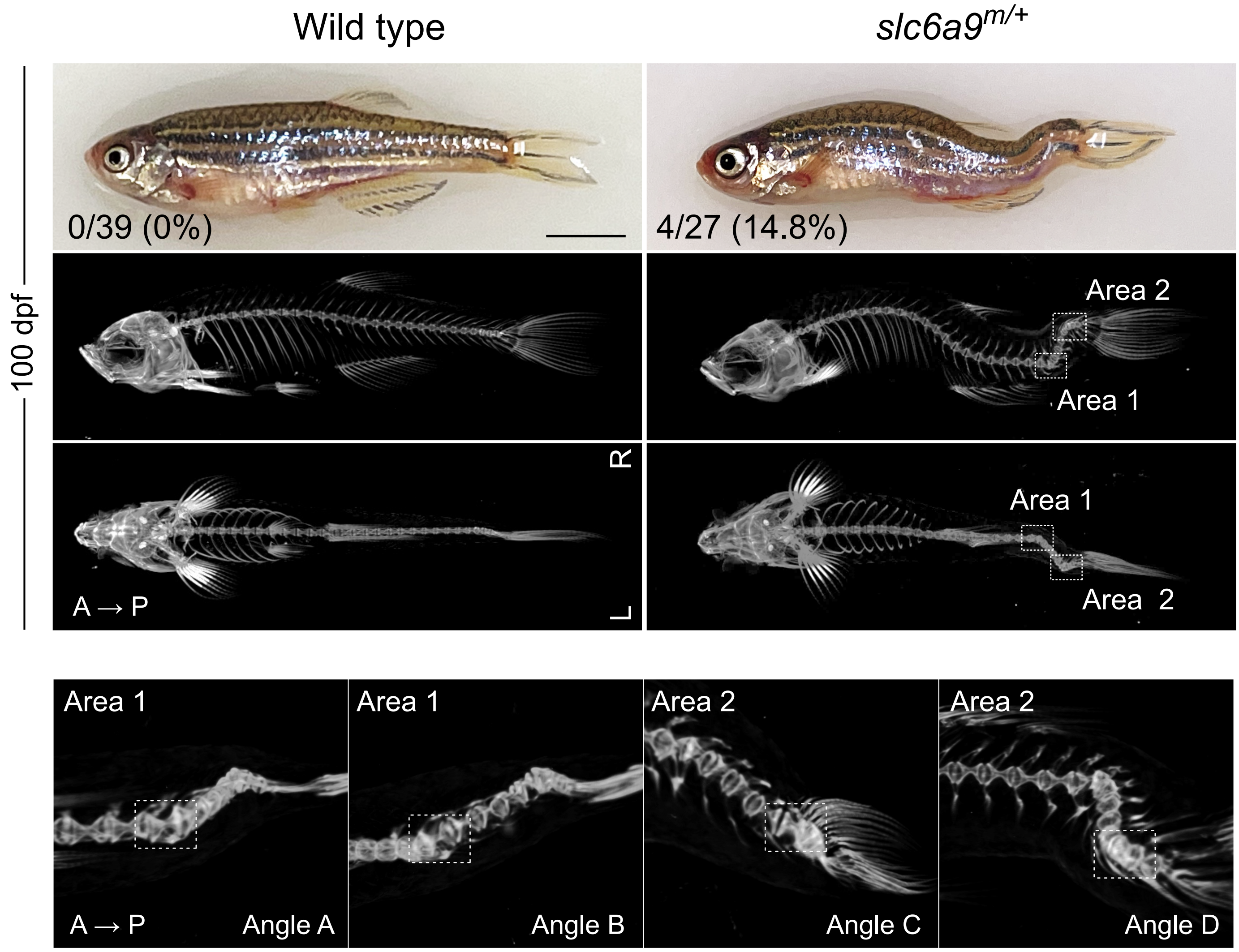

Supplemental Figure 11. Spinal curvature in adult *slc6a9* mutant zebrafish. Spinal phenotype and micro-CT images of wild type and *slc6a9*$^{m/+}$ zebrafish at adult stage (100 dpf). Images are shown in either side or dorsal view. A, anterior; P, posterior; L, left; R, right. Scale bar indicates 0.5 cm. The number of analyzed fish and the penetrance of spinal curvature are indicated for each genotype. To detect the details of apices of curvatures, the two curvature regions (Area 1 and 2) of *slc6a9*$^{m/+}$ zebrafish are enlarged and oriented in different angles (lower panel). The adjacent vertebrae that form apices are labeled. Noted that all the highlighted vertebrae are morphologically normal.

Supplemental Figure 12

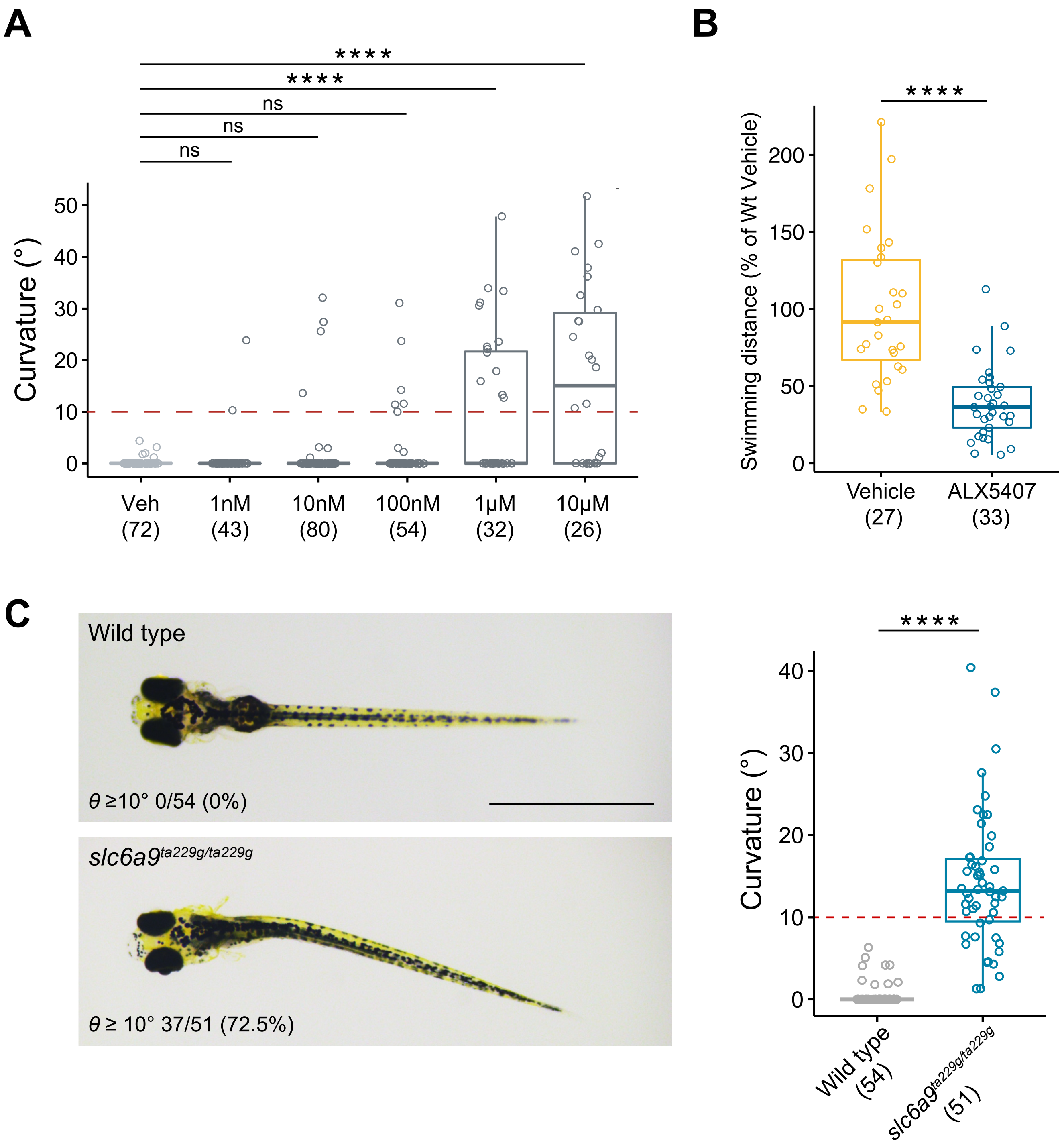

Supplemental Figure 12. Phenotype of wild type zebrafish by ALX5407 administration and *slc6a9 ta229g* mutant line. (**A**) Results of ALX5407 treatment on wild type larvae. Larvae were administrated with different dosages of ALX5407 from 2 dpf to 7 dpf. The number of analyzed fish is indicated on the bottom of each box. Each data point represents the axial curvature degree (θ) of an individual larva. Noted that there are 0%, 4.65%, 5.0%, 9.26%, 40.63% and 57.69% of wild type larvae acquiring axial curvature ($\theta \geq 10°$) from treatment of vehicle, 1 nM, 10 nM, 100 nM, 1 μM and 10 μM ALX5407, respectively. 1-way ANOVA test, **** $P < 0.0001$. (**B**) Quantification of swimming distances of wild type larvae treated with vehicle (n = 27) or 1 μM ALX5407 (n = 33). Each data point represents the swimming distance of an individual larva. Unpaired Student's *t*-test, ****$P < 0.0001$; ns, no significance. (**C**) Axial curvature of *slc6a9*$^{ta229g/ta229g}$ mutant zebrafish at 7 dpf. The number of analyzed fish and the penetrance of curvature ($\theta \geq 10°$) are quantified and indicated for each genotype. The scale bar indicates 1 mm. Unpaired Student's *t*-test, **** $P < 0.0001$. In all charts, boxes show the median and IQRs with all individual data points superimposed.

Supplemental Figure 13

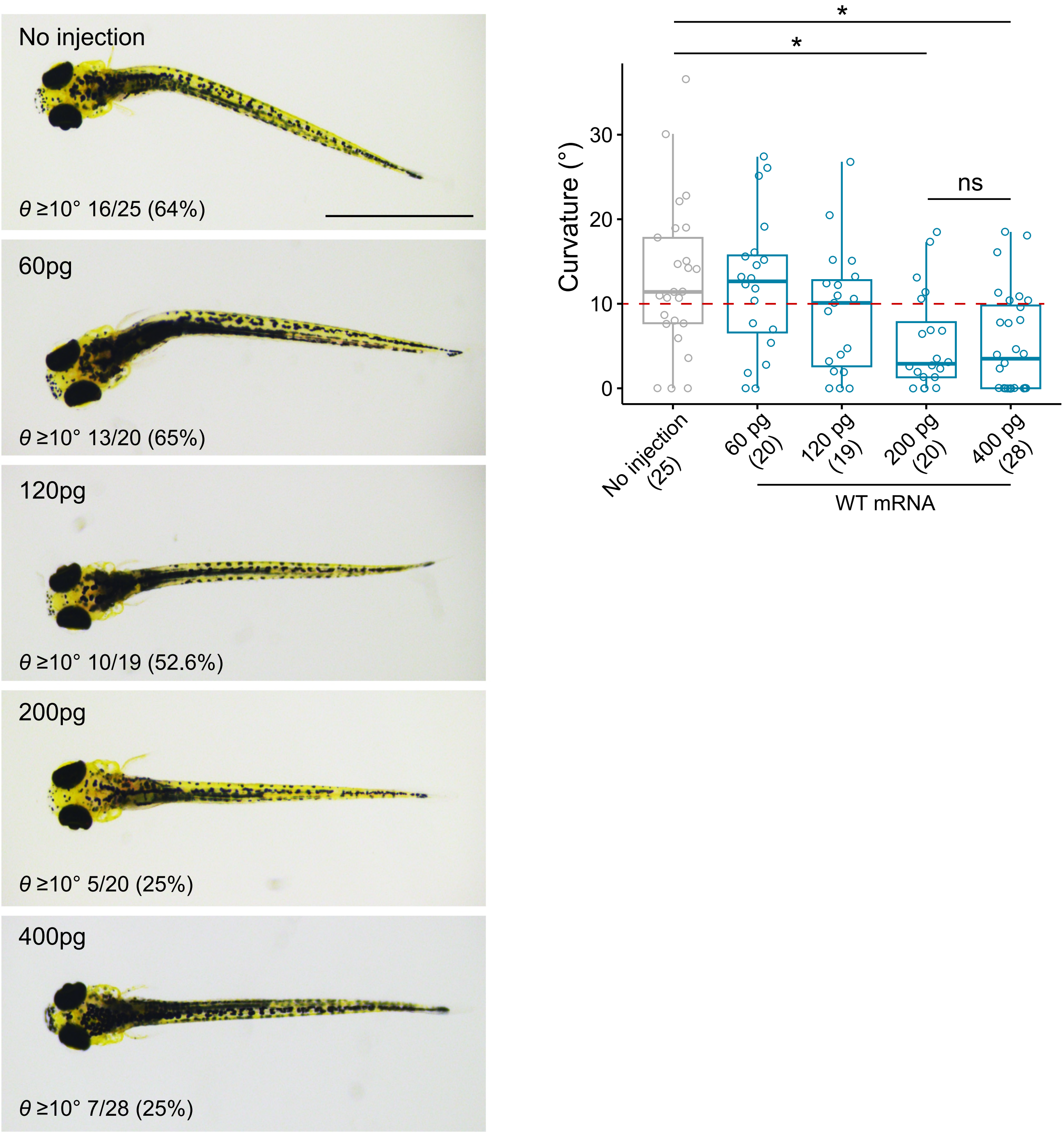

Supplemental Figure 13. Dosage dependent rescue of axial curvature by microinjection of *GLYT1* wild type mRNA in *slc6a9*$^{m/m}$ zebrafish. 60, 120, 200 and 400 pg wild type *GLYT1* mRNA were injected in zygotes generated from *slc6a9*$^{m/+}$ and *slc6a9*$^{m/+}$ mating pairs. The number of analyzed *slc6a9*$^{m/m}$ fish is indicated on the bottom of each box. Each data point represents the axial curvature degree (θ) of an individual larva. Boxes show the median and IQRs with all individual data points superimposed. 1-way ANOVA test, $*P < 0.05$; ns, no significance.

Supplemental Figure 14

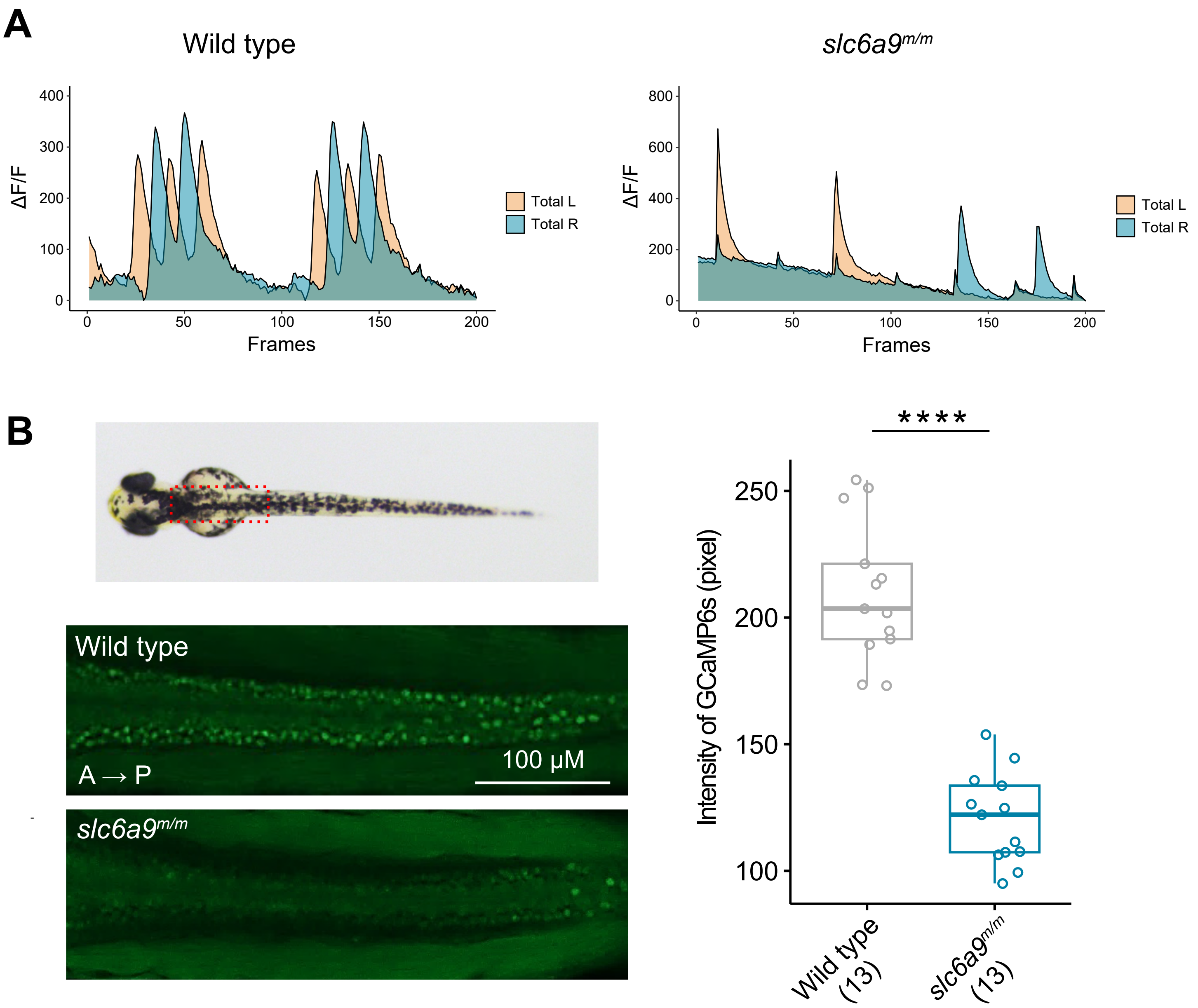

Supplemental Figure 14. Quantification of neural activities and activated neurons in wild type and *slc6a9* mutant zebrafish. (A) Quantification of the GCaMP6s fluorescent signals in both sides of body in wild type and *slc6a9*$^{m/m}$ zebrafish larvae in a *Tg(elavl3-H2B-GCaMP6s)* background at 24 hpf. Each frame was taken with a 100-ms exposure and at 10 fps. The intensity of fluorescent signals from total left- or right-side body intensity were analyzed and normalized. The GCaMP6s fluorescence intensity was defined as the ΔF/F, and ΔF/F changes within 20 seconds recording time were shown. **(B)** Quantification of the fixed GCaMP6s fluorescent signals in a dorsal view in wild type and *slc6a9*$^{m/m}$ zebrafish larvae in a *Tg(elavl3-H2B-GCaMP6s)* background at 24 hpf. The intensity of fluorescent signals from the dorsal snap captures were analyzed. Each data point represents the GCaMP6s intensity of an individual larva. Boxes show the median and IQRs with all individual data points superimposed. Unpaired Student's *t*-test, ****$P < 0.0001$. A, anterior; P, posterior.

Supplemental Figure 15

A

Wild type	c.	cgc	tgc	agg	aac	cac	ggg	gtg	ctg	tcc	tgg	ctg	aaa	ggg	cac	aag	cgc	tac	tgc	cga
	p.	R	C	R	N	H	G	V	L	S	W	L	K	G	H	K	R	Y	C	R
		30	31	32	33	34	35	36	37	38	39	40	41	42	43	44	45	46	47	48
*dmrt3a^m/m^*	c.	cgc	tgc	agg	aat	gct	gtc	ctg	gct	gaa	agg	gca	caa	gcg	cta	ctg	ccg	att	taa	
	p.	R	C	R	N	A	V	L	A	E	R	A	Q	A	L	L	P	I	*	

B

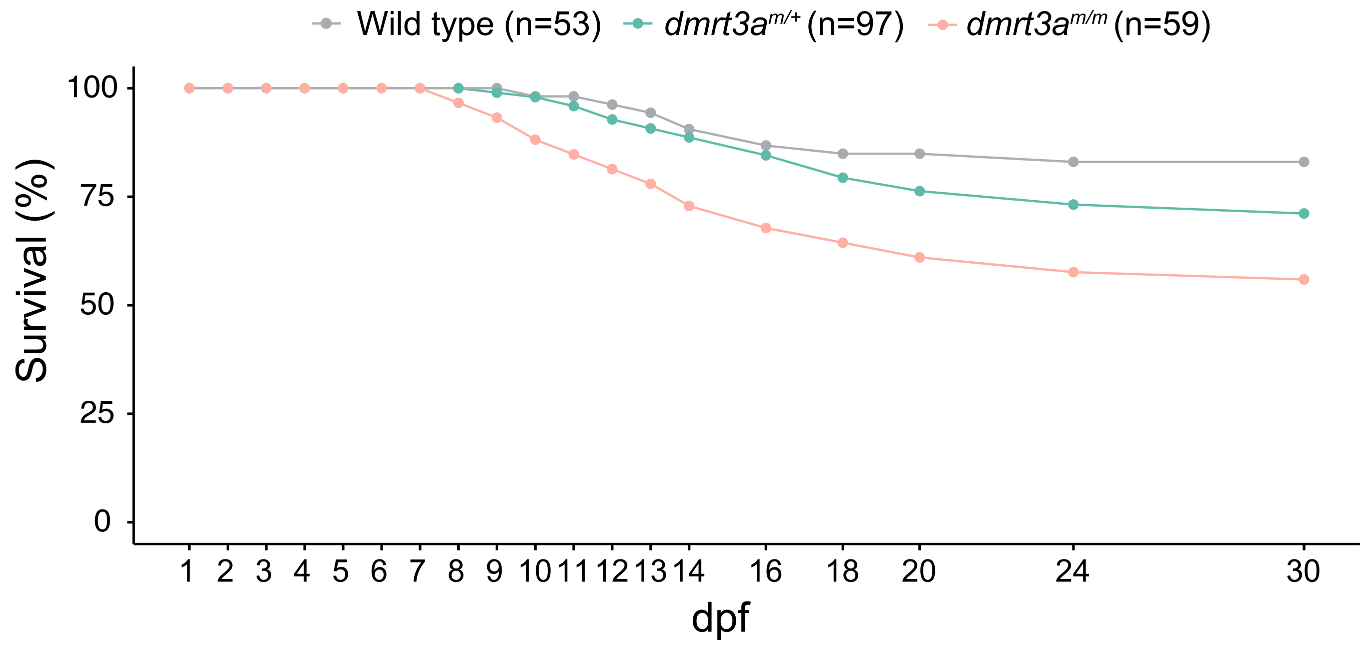

C

18 dpf

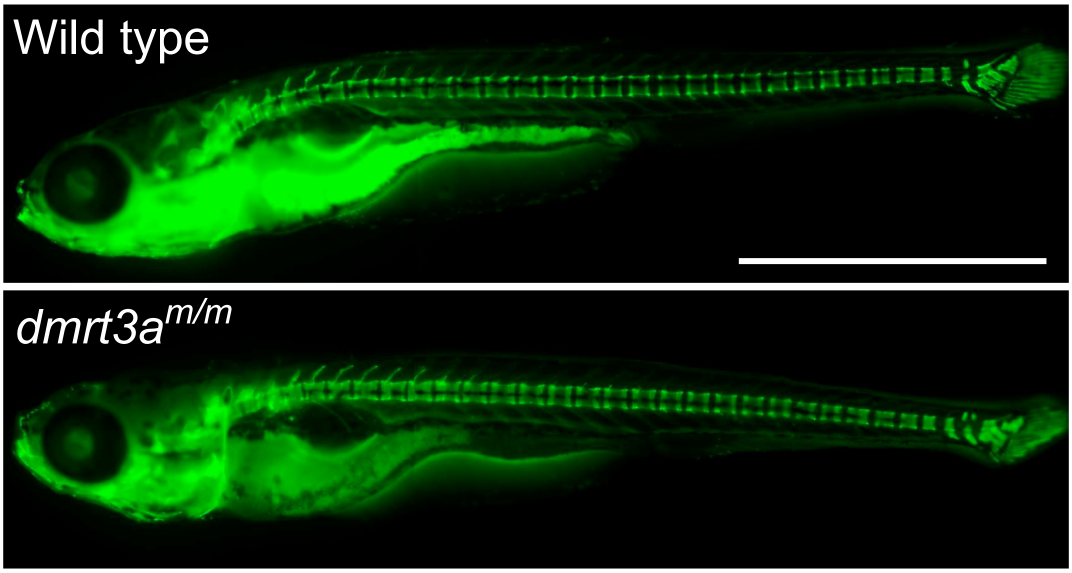

Supplemental Figure 15. Generation and phenotype of the *dmrt3a* mutant zebrafish model. (A) The 8 base pairs (bp) deletion of dmrt3a results in a frameshift and truncated dmrt3a in zebrafish. The 8bp deletion is highlighted in red. **(B)** The survival curve of wild type and *dmrt3a* mutant zebrafish within 30 days after fertilization. **(C)** Live imaging of 18 dpf wild type and *dmrt3a*$^{m/m}$ zebrafish stained with calcein (green). Scale bar indicates 2 mm.

Supplemental Figure 16

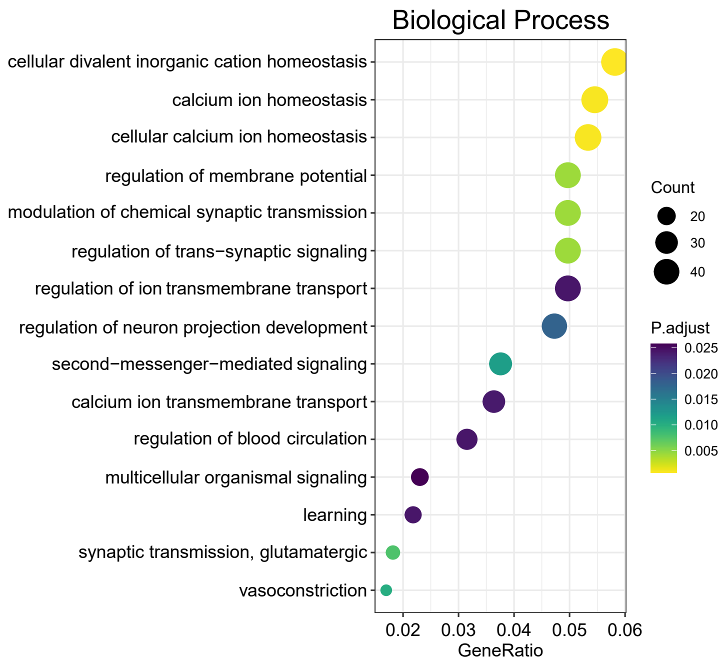

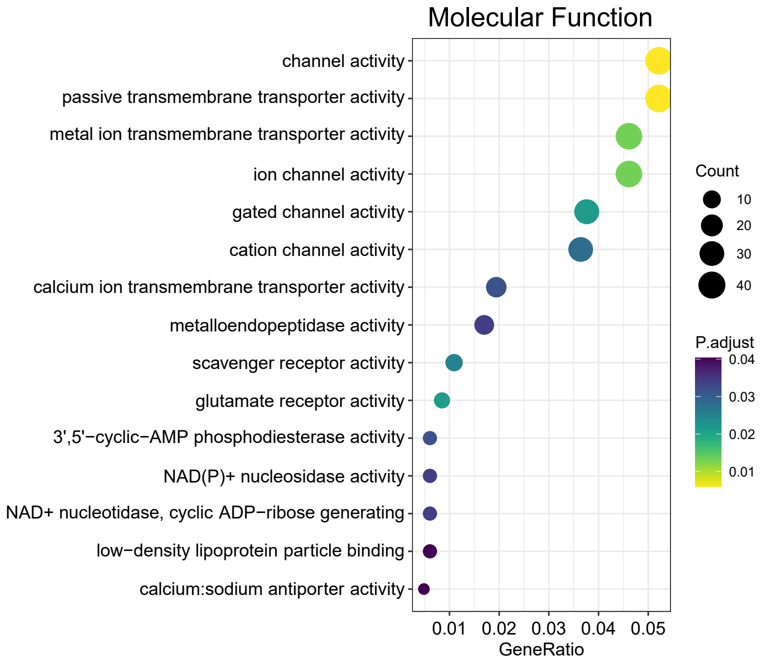

Supplemental Figure 16. Gene Ontology (GO) functional enrichment analysis of AIS associated genes. Two categories of GO terms including Biological Process and Molecular Function of AIS associated genes are shown. GeneRatio is the ratio of genes mapped to a pathway to the total gene set. The size of the dots represents the number of genes mapped to the pathway.
